# A Comparison of Acute Toxicity Endpoints for Adult Honey Bees with Technical Grade Active Ingredients and Typical End-use Products as Test Substance

**DOI:** 10.1093/jee/toz305

**Published:** 2019-11-22

**Authors:** Susan E Spruill, Bridget F O'Neill, Silvia Hinarejos, Ana R Cabrera

**Affiliations:** 1 Applied Statistics and Consulting, Spruce Pine NC; 2 Corteva Agriscience, Indianapolis, IN; 3 AgroSolutions Division International, Sumitomo Chemical, 10A Rue de la Voie Lactée, Saint Didier au Mont d’Or, France; 4 Environmental Safety, Bayer CropScience LP, Chesterfield, MO

**Keywords:** *Apis mellifera*, pesticide, contact toxicity, oral toxicity, median lethal dose

## Abstract

The honey bee, *Apis mellifera* L. (Hymenoptera: Apidae), is a model organism for pollinators in risk assessment frameworks globally. The acute toxicity tests with adult honey bees for contact and oral exposure are part of the requirements for pesticide registration and are typically conducted with the active ingredient. A question often asked is if the typical end-use product (TEP) is more toxic than the technical grade active ingredient (TGAI) to honey bees. We explored this question by mining publicly available databases from regulatory agencies worldwide, where testing with the TEP is required. The objective of this study was to determine whether TEPs are comparable in toxicity to the TGAI. The dataset was analyzed via a 3 × 3 contingency table with toxicity categories, as the data cannot be computed for regression analysis. Of the 151 active ingredients with reported endpoints for contact exposure, 28 were classified as either moderately or highly toxic, 123 were classified as practically nontoxic, and 3 were inconclusive. Only two (1.3%) were reclassified from nontoxic to moderately toxic as the TEP. Of the 141 active ingredients with reported endpoints for oral exposure, 23 were classified as moderately or highly toxic, 113 were classified as practically nontoxic, and 5 were inconclusive. Only five (3.6%) were reclassified from nontoxic to moderately toxic as the TEP. Fewer than 5% of the total TEPs evaluated (contact and oral) were shown to be more toxic than the TGAI, suggesting that the risk assessments of TGAIs would be sufficiently protective to pollinators at the screening laboratory level.

The honey bee, *Apis mellifera* L. (Hymenoptera: Apidae), serves as a model organism for pollinators in the current risk assessment framework in North America and other regions of the world (EFSA 2013, USEPA, PMRA, and CDPR 2014, USEPA 2016). The acute toxicity tests with adult honey bees for contact and oral exposure are part of the current requirements for pesticide registration. Acute toxicity endpoints are the medial lethal doses (LD_50_) and are expressed as µg active ingredient/bee. These tests are required and typically conducted with the technical grade active ingredient (TGAI), however in some cases, a typical end-use product (TEP; formulated product) may be needed in addition to data on TGAI if there are data indicating that a TEP is potentially more toxic than the TGAI, and bees may be directly exposed to the intact TEP (USEPA, PMRA, and CDPR 2014). Furthermore, sometimes the TEP is used to overcome solubility limits with the TGAI in the bee testing diet (sucrose solution) for the oral acute test.

A recurring question has been if the TEP can be more toxic than the TGAI to honey bees (Mullin et al. 2015). We seek to explore this question by mining publicly available databases from regulatory agencies in North America and Europe. These databases were 1) the U.S. Environmental Protection Agency (US EPA) EcoTox Knowledgebase (https://cfpub.epa.gov/ecotox/), 2) the European Food Safety Authority (EFSA) Register of Questions (http://registerofquestions.efsa.europa.eu/roqFrontend/login?5), 3) the EU Commission Pesticide Database (http://ec.europa.eu/food/plant/pesticides/eu-pesticides-database/public/?event=homepage&language=EN), and 4) the National Institute for Agricultural Research of France AGRITOX Database (http://www.agritox.anses.fr/index2.php).

The objectives of this study were 1) to determine whether TEPs are either more or less toxic than the TGAI, 2) to determine whether there are any specific TEP types or chemical classes that result in higher toxicity to adult honey bees than the TGAI, and 3) to determine whether findings differ for contact versus oral exposure.

## Materials and Methods

The reported LD_50_ values of 252 compounds were retrieved for this analysis and categorized if the endpoint was generated with a TGAI or TEP, and if exposure route was via contact and oral. Some limitations of this approach are that background information was not available regarding the variability of the reported endpoints (e.g., confidence intervals), it was unclear if the study design followed guidelines and Good Laboratory Practices (OECD 1998), or if there were solubility barriers of the molecule that dictated the selection of test levels. However, the reported LD_50_ values have been used to approve the registration of TEPs and as such, these values must meet certain quality criteria set by each regulatory agency. Therefore, these values were considered acceptable for this exploratory analysis.

The compounds were grouped according to the type of pesticide (fungicide, herbicide, insecticide) and type of TEP (formulation). If more than one LD_50_ value was found for the TGAI, the lowest was used for this analysis. Due to the low representation of certain types of TEPs, and to enhance the number for comparisons, they were grouped into ‘super-TEP’ categories based on shared physical characteristics (e.g., solids, liquids), as outlined in the Australian Pesticide and Veterinary Medicines Authority website (https://apvma.gov.au/node/10901).

The large proportion of contact and oral LD_50_s reported as an inequality (i.e., >100 µg a.i./bee) for one or both of the test materials (TGAI or TEP) prevented a meaningful representation of the dataset for conducting a regression analysis, thus leading us to evaluate the data via a 3 × 3 contingency table with toxicity categories. Historically, the USEPA has classified compounds based on the acute contact endpoint in three categories: 1) highly toxic if the LD_50_ is <2 µg/bee, 2) moderately toxic if LD_50_ is between 2 and 11 µg/bee, and 3) practically nontoxic if the LD_50_ is >11 µg/bee (USEPA, PMRA, and CDPR 2014). USEPA has not extended this classification scheme to include acute oral endpoints for regulatory purposes, but for our analysis we extended this scheme to the oral LD_50_ values to maintain consistency in our approach. In instances where the LD_50_ for both TGAI and TEP were reported as inequalities and were below 11 µg/bee, the comparison was classified as ‘Inconclusive’. If more than one TEP value was available with the same TGAI, each was classified individually. Compounds with no TEP or LD_50_ data were culled from the analysis dataset. The toxicity classifications served as the basis for a two-way cross tabulation to assess concordance of TEP toxicity against TGAI. The proportions of cases where the TEP toxicity classification were worse than the TGAI classification, were compared between contact and oral routes of exposure with a Fisher’s Exact Test using SAS statistical analysis software (V9.4 for PC, www.sas.com).

## Results

We identified cases where the toxicity classification of the TEPs was different compared to the TGAI. Of the 151 active ingredients with reported LD_50_ for contact exposure, 28 (18.5%) were classified as either moderately (*n* = 5) or highly toxic (*n* = 23) (Table 1). All reported highly toxic ingredients were insecticides, and predominantly of the pyrethroid chemical class (*n* = 12; 8%). The remaining highly toxic ingredients were evenly distributed among carbamates (*n* = 2), neonicotinoids (*n* = 2), organophosphates (*n* = 2), macrocyclic lactones (*n* = 2), or unclassified (*n* = 3).

**Table 1.** Cross-tabulations of toxicity classification of TEPs and TGAI for CONTACT exposure data (*n* = 151)

Toxicity classification based on TEP LD_50_	Toxicity classification based on TGAI LD_50_*N* (%)			
	Highly toxic (<2 µg/bee)	Moderately toxic (2–11 µg/bee)	Practically nontoxic (>11 µg/bee)	Inconclusive
Highly toxic	20 (13.3%)	0	0	0
Moderately toxic	1 (0.7%)	1 (0.7%)	2 (1.3%)	0
Practically nontoxic	1 (0.7%)	4 (2.7%)	116 (76.8%)	3 (2%)
Inconclusive	1 (0.7%)	0	2 (1.3%)	0

The values below represent the number (percentage) of compounds on the same or different toxicity category based on the LD_50_ derived with the TGAI or with the TEP.

The proportion of active ingredients with reported LD_50_ for oral exposure was similar with 23 of 141 (16.3%) being either moderately toxic (*n* = 4) or highly toxic (*n* = 19) (Table 2). Like the contact exposure, all oral exposure ingredients classified as highly toxic were insecticides and were predominantly pyrethroids (*n* = 9; 6.4%). Few active ingredients had inconclusive toxicities: three (2%) in the contact exposure and five (3.5%) in the oral exposure. The remaining TGAIs were classified as practically nontoxic: 120 (79.5%) for contact exposure and 113 (80%) for oral exposure.

**Table 2.** Cross-tabulations of toxicity classification of TEPs and TGAI for ORAL exposure data (*n* = 141)

Toxicity classification based on TEP LD_50_	Toxicity classification based on TGAI LD_50_*N* (%)			
	Highly toxic (< 2 µg/bee)	Moderately toxic (2 to 11 µg/bee)	Practically nontoxic (>11 µg/bee)	Inconclusive
Highly toxic	14 (9.9%)	2 (1.4%)	0	0
Moderately toxic	3 (2.1%)	0	5 (3.6%)	0
Practically nontoxic	1 (0.7%)	2 (1.4%)	107 (75.9%)	5 (3.6%)
Inconclusive	1 (0.7%)	0	1 (0.7%)	0

The values below represent the number (percentage) of compounds on the same or different toxicity category based on the LD_50_ derived with the TGAI or with the TEP.

With regards to contact exposure, only two (1.3%) TEPs were classified as being more toxic than the TGAI, while six (4%) TEPs were classified as being less toxic than the TGAI. With regards to oral exposure, seven (5%) TEPs were classified as more toxic than the TGAI while seven (5%) were classified as being less toxic that the TGAI. Using Fisher’s Exact test of proportions, the proportion of TEPs that increased in toxicity were not found to be statistically significantly different based on routes of exposure (two-sided Pr≤*P*; *P* = 0.0942). Among the active ingredients with TEPs that were more toxic, the majority were formulated as emulsifiable concentrates which represented the most common formulation in the compiled databases. No patterns could be established for chemical classes due to lack of representation in the database.

## Discussion

The compiled contact and oral pesticide toxicity data are by no means a complete assembly of chemical compounds and all TEPs. Nonetheless, the datasets are an unbiased attempted at addressing the broad question of how the toxicity of TEPs to honey bee adults compare with the TGAI. The datasets covered a wide range of chemical compounds with nearly equal representation for the type of pesticide based on the target organisms they control: herbicides, fungicides and insecticides. Both wet (e.g., emulsifiable concentrate) and dry (e.g., wettable powder) TEPs were represented with approximately 70% being wet in both contract and oral data sets. The most common TEP type was emulsifiable concentrates. Of the highly toxic active ingredients, all were insecticides, and most were in the pyrethroids chemical family. In general, the TEPs toxicity classifications were largely the same as their TGAI toxicity classifications, suggesting the laboratory level assessments of TGAIs would be sufficiently protective in the majority of cases. In addition, this also indicates that to overcome solubility limits of the TGAI for an oral acute test, endpoints generated with a TEP or other formulations are representative and valid. While there were a few instances where the TEP was classified as more toxic than the TGAI, the overall result from this analysis suggest that the current risk assessment process in North America is largely protective in the majority of registrations. In those few instances, we were not able to identify trends regarding chemical class or formulation type. Higher tier testing (i.e., tunnel studies, field studies) is conducted with TEPs, so these studies will account for any toxicity difference for pesticides that don’t pass the laboratory level or are highly toxic to bees.

## Supplementary Material

toz305_suppl_Supplemental_Information
