# Supplementary material for: A Comparison of Acute Toxicity Endpoints for Adult Honey Bees with Technical Grade Active Ingredients and Typical End-use Products as Test Substance
Source: J Econ Entomol. 2019 Nov 22;113(2):1015–7. doi: 10.1093/jee/toz305 (PMC7136193; doi:10.1093/jee/toz305)
Supplement: toz305_suppl_Supplemental_Information [file toz305_suppl_supplemental_information.docx]

Supplemental Information

**A comparison of acute toxicity endpoints for adult honey bees with technical grade active ingredients and typical end-use products as test substance**

Susan E. Spruill^1^, Bridget O’Neill^2^, Silvia Hinarejos ^3^, Ana R. Cabrera^4*^

^1^ Applied Statistics and Consulting, 1205 Chestnut Mountain Rd, Spruce Pine NC 28777

^2^ Corteva Agriscience, 9330 Zionsville Road, Indianapolis, IN 46268, USA

^3^ Sumitomo Chemical, 10A Rue de la Voie Lactée, Saint Didier au Mont d’Or 69370, France

^4^ Bayer CropScience LP, 2 TW Alexander Dr., Research Triangle Park NC 27709

*Corresponding author: Ana R. Cabrera, [ana.cabrera@bayer.com](mailto:ana.cabrera@bayer.com), +1 (919) 884-9843

Supplemental Table 1. List of all Technical Grade Active Ingredients (TGAI) in alphabetical order, obtained from four publicly available databases to mine the honey bee contact acute endpoint (Lethal median dose or LD_50_, expressed as µg a.i./bee). The four databases were the US Environmental Protection Agency (A), the European Food Safety Authority (B), the European Commission Pesticides Database (C), and the National Institute for Agricultural Research of France (D). The pesticide type, chemical class, type of Typical End-use Product (TEP), Super-TEP category (based on a broader description of the physical characteristics of the formulated product), and toxicity classification for each TGAI are identified.

| **TGAI** | **Pesticide Type** | **Chemical Class** | **Mined TGAI LD_50_ (µg a.i./bee)** | **TGAI**  **Toxicity Classification** | **TEP Type** | **Super -TEP** | **Mined TEP LD_50_ (µg a.i./bee)** | **TEP Toxicity Classification** |
| --- | --- | --- | --- | --- | --- | --- | --- | --- |
| Abamectin | Insecticide | Avermectins | 0.0022 (D),  0.41 (A) | Highly Toxic |  |  |  | No Data |
| Acequinocyl | Miticide | Quinoline | >100 (A, D),  >350 (A) | Nontoxic | Aqueous Suspension | Liquid Suspensions | >53.9 [Kanemite] (D) | Nontoxic |
| Acetamiprid | Insecticide | Neonicotinoid | 8.1 (A, C) | Moderately Toxic | Concentrate | Liquid Solutions | 9.29 [20% C] (C) | Moderately Toxic |
| Acibenzolar-S- methyl (benzothiadiazole) | Fungicide | Benzothiadiazole | >100 (C, D) | Nontoxic | Water-dispersible Granules | Solid for Direct Use | >200 [Bion 50WG] (D) | Nontoxic |
| Aclonifen | Herbicide | Diphenyl ether | >100 (D) | Nontoxic | Aqueous Suspension | Liquid Suspensions | >205 [Bandur 600SC] (D) | Nontoxic |
| Acrinathrin | Miticide | Pyrethroid | 0.084 (D) | Highly Toxic |  |  |  | No Data |
| Alphamethrin (alpha cypermethrin) | Insecticide | Pyrethroid | 0.033 (C, D) | Highly Toxic | Emulsifiable Concentrate | Liquid Solutions | 0.11 [100g/L,  Fastac] (C, D) | Highly Toxic |
| Ametoctradin | Fungicide | Triazolopyrimidine | >100 (D) | Nontoxic | Flowable (liquid) | Liquid Solutions | >57.7 [BAS65100F,  mixture of ametoctradine and dimethomorph] (D) | Nontoxic |
| Amitrole (aminotriazole) | Herbicide | Triazole | >100 (C,),  >200 (A) | Nontoxic |  |  |  | No Data |
| Azoxystrobin | Fungicide | Strobin | >200 (A, D) | Nontoxic | Water-dispersible Granules | Solid for Direct Use | >200 [Amistar500] (D) | Nontoxic |
| Azoxystrobin | Fungicide | Strobin | >200 (A, D) | Nontoxic | Aqueous Suspension | Liquid Suspensions | >200 [Amistar 250] (D) | Nontoxic |
| Beflubutamid | Herbicide | Amide | >200 (C, D) | Nontoxic |  |  |  | No Data |
| Benalaxyl | Fungicide | Xylylalanine |  | No Data |  |  |  | No Data |

| **TGAI** | **Pesticide Type** | **Chemical Class** | **Mined TGAI LD_50_ (µg a.i./bee)** | **TGAI**  **Toxicity Classification** | **TEP Type** | **Super -TEP** | **Mined TEP LD_50_ (µg a.i./bee)** | **TEP Toxicity Classification** |
| --- | --- | --- | --- | --- | --- | --- | --- | --- |
| Benfluralin | Herbicide | 2,6-Dinitroaniline | 14.5, >101  (A), >100 (D) | Nontoxic | Emulsifiable Concentrate | Liquid Solutions | >100 [Bonalan, 182g/L] (D) | Nontoxic |
| Benoxacor | Herbicide safener | Unknown | >100 (D) | Nontoxic |  |  |  | No Data |
| Bentazone | Herbicide | Unclassified | >200 (C, D) | Nontoxic |  |  |  | No Data |
| Benthiavalicarb- Isopropyl | Fungicide | Carbamate | >100 (D) | Nontoxic | Water-dispersible Granules | Solid for Direct Use | >140 [WG1.75%] (D) | Nontoxic |
| Betacyfluthrin | Insecticide | Pyrethroid | 0.037, 0.005,  0.0677 (A) ,  0.0098 (C,D) | Highly Toxic |  |  |  | No Data |
| Bifenazate | Miticide | Hydrazine carboxylate | 7.8, (A), 8.5 (C, D)  [Geomean 8.14] | Moderately Toxic | Aqueous Suspension | Liquid Suspensions | >98 [480g/L] (C, D) | Nontoxic |
| Bifenox | Herbicide | Diphenyl ether | >200 (D) | Nontoxic |  |  |  | No Data |
| Bifenthrin | Insecticide | Pyrethroid | 0.015 (A, D) | Highly Toxic | Aqueous Suspension | Liquid Suspensions | 0.0016  [Talstar8SC] (D) | Highly Toxic |
| Boscalid | Fungicide | Carboxamide | >200 (A, C, D) | Nontoxic | Water-dispersible Granules | Solid for Direct Use | >100  [Cantus50%WG] (D) | Nontoxic |
| Bromoxynil octanoate | Herbicide | Hydroxybenzonitrile | 14.5 (A),>100 (D) | Nontoxic |  |  |  | No Data |
| Bromoxynil phenol | Herbicide | Hydroxybenzonitrile | 150 (D) | Nontoxic |  |  |  | No Data |
| Bromuconazole | Fungicide | Azole | >100 (D) | Nontoxic |  |  |  | No Data |
| Bupirimate | Fungicide | Pyrimidine | >50 (D) | Nontoxic |  |  |  | No Data |

| **TGAI** | **Pesticide Type** | **Chemical Class** | **Mined TGAI LD_50_ (µg a.i./bee)** | **TGAI**  **Toxicity Classification** | **TEP Type** | **Super -TEP** | **Mined TEP LD_50_ (µg a.i./bee)** | **TEP Toxicity Classification** |
| --- | --- | --- | --- | --- | --- | --- | --- | --- |
| Captan | Fungicide | Thiophthalimide | >215 (A),  >200 (D) | Nontoxic | Water-dispersible Granules | Solid for Direct Use | >200 [Merpan 80WDG] (D) | Nontoxic |
| Carbetamide | Herbicide | Amide |  | No Data | Water-dispersible Granules | Solid for Direct Use | >100 [WG60%] (D) | Nontoxic |
| Carboxin | Fungicide | Carboxamide | 181 (A), >100 (D) | Nontoxic |  |  |  | No Data |
| Carfentrazone ethyl | Herbicide | Triazolone | >200 (D) | Nontoxic |  |  |  | No Data |
| Chlorantraniliprole | Insecticide | Anthranilic diamide | >4 (D) | Inconclusive | Water-dispersible Granules | Solid for Direct Use | >100 [DPX-E2Y45 35WG] (D) | Nontoxic |
| Chlorantraniliprole | Insecticide | Anthranilic diamide | >4 (D) | Inconclusive | Aqueous Suspension | Liquid Suspensions | >100 [DPX-E2Y45 20SC] (D) | Nontoxic |
| Chloridazone | Herbicide | Pyridazinone | >200 (D) | Nontoxic | Water-dispersible Granules | Solid for Direct Use | >130 [>200µg product/bee, Pyramin WG, 650 g/kg] (D) | Nontoxic |
| Chlorothalonil | Fungicide | Substituted Benzene | 181.29 (A),  >63 (D) | Nontoxic |  |  |  | No Data |
| Chlorotoluron | Herbicide | Urea |  | No Data | Aqueous Suspension | Liquid Suspensions | >119.3 [700g/L] (D) | Nontoxic |
| Chlorpyriphos | Insecticide | Organophosphate | 0.32, 0.068,  0.01, 0.114 (A) | Highly Toxic |  |  |  | No Data |
| Chlorpyriphos- ethyl | Insecticide | Organophosphate | 0.059 (D) | Highly Toxic |  |  |  | No Data |
| Chlorpyriphos- methyl | Insecticide | Organophosphate | 0.15 (D) | Highly Toxic | Emulsifiable Concentrate | Liquid Solutions | 0.15 [225 g/L] (D) | Highly Toxic |
| Clodinafop- propargyl | Herbicide | Aryloxyphenoxy propionic acid | >100 (A) | Nontoxic | Emulsifiable Concentrate | Liquid Solutions | 40.9 [100g/L,  Topik] (D) | Nontoxic |
| Clomazone | Herbicide | Unclassified | >100 (A, D) | Nontoxic |  |  |  | No Data |

| **TGAI** | **Pesticide Type** | **Chemical Class** | **Mined TGAI LD_50_ (µg a.i./bee)** | **TGAI**  **Toxicity Classification** | **TEP Type** | **Super -TEP** | **Mined TEP LD_50_ (µg a.i./bee)** | **TEP Toxicity Classification** |
| --- | --- | --- | --- | --- | --- | --- | --- | --- |
| Clopyralid | Herbicide | Pyridenecarboxylic acid | >100 (A),  >98.1 (D) | Nontoxic |  |  |  | No Data |
| Clothianidin | Insecticide | Neonicotinoid | 0.0218,  0.0439 (A),  0.04426,  0.0389 (C, D),  0.0275 | Highly Toxic |  |  |  | No Data |
| Cyazofamid | Fungicide | Azole | >100 (A, D) | Nontoxic | Aqueous Suspension | Liquid Suspensions | >100 [Ranman SC400] (D) | Nontoxic |
| Cyfluthrin | Insecticide | Pyrethroid | 0.037, 0.067,  0.005 (A) [Geomean 0.023] | Highly Toxic | Emulsifiable Concentrate | Liquid Solutions | 0.0098 [50g/L,  Baythroid] (D) | Highly Toxic |
| Cyhalofop-butyl | Herbicide | Aryloxyphenoxy propionic acid | >100 (A, D) | Nontoxic | Emulsifiable Concentrate | Liquid Solutions | >100 [Clincher EC200] (D) | Nontoxic |
| Cymoxanil | Fungicide | Cyanoacetamide oxime | >25 (A), >100 (D) | Nontoxic | Water-dispersible Granules | Solid for Direct Use | 60 [with Famoxadone 52.5WG] (D) | Nontoxic |
| Cypermethrin | Insecticide | Pyrethroid | 0.097, 0.169,  0.107, 0.023  (A), 0.02 (C, D) [Geomean 0.06] | Highly Toxic | Emulsifiable Concentrate | Liquid Solutions | 0.88 [100EC] (C, D), 0.031 [400EC] (D) | Highly Toxic |
| Cyproconazole | Fungicide | Azole | >100 (A, D) | Nontoxic | Soluble Concentrate | Liquid Simple Solutions | 13 [Alto100SL] (D) | Nontoxic |
| Cyprodinil | Fungicide | Pyrimidine | 396 (A), >784 (A, D) | Nontoxic | Water-dispersible Granules | Solid for Direct Use | >75 [Unix75WG],  >125  [Chorus50WG] (D) | Nontoxic |

| **TGAI** | **Pesticide Type** | **Chemical Class** | **Mined TGAI LD_50_ (µg a.i./bee)** | **TGAI**  **Toxicity Classification** | **TEP Type** | **Super -TEP** | **Mined TEP LD_50_ (µg a.i./bee)** | **TEP Toxicity Classification** |
| --- | --- | --- | --- | --- | --- | --- | --- | --- |
| Deltamethrin | Insecticide | Pyrethroid | 0.667, 0.067  (A), 0.0015  (A, D)  [Geomean 0.04] | Highly Toxic | Emulsifiable Concentrate | Liquid Solutions | 0.010 [Decis25EC] (D) | Highly Toxic |
| Desmedipham | Herbicide | Carbamate | >50 (A, D) | Nontoxic | Emulsifiable Concentrate | Liquid Solutions | 54.2 [160g/L +  160g/L PMP,  Betanal AM11] (D) | Nontoxic |
| Dicamba | Herbicide | Benzoid Acid | >90.65 (A),  >100 (D) | Nontoxic | Soluble Concentrate | Liquid Simple Solutions | >100 [Banvel 480SL] (D) | Nontoxic |
| Dichlorprop P | Herbicide | Chlorophenoxy acid | >200 (D) | Nontoxic |  |  |  | No Data |
| Diclofop methyl | Herbicide | Aryloxyphenoxy propionic acid | >100 (D) | Nontoxic |  |  |  | No Data |
| Diethofencarb | Fungicide | Carbamate | >100 (D) | Nontoxic | Wettable Powders | Solid for Direct Use | >100 [Powmyl 25WP] (D) | Nontoxic |
| Difenoconazole | Fungicide | Azole | >101 (A),  >100 (A, D) | Nontoxic |  |  |  | No Data |
| Diflubenzuron | Insecticide | Benzoylurea | >114.8 (A),  >100 (D) | Nontoxic |  |  |  | No Data |
| Diflufenican | Herbicide | Carboxamide | >100 (D) | Nontoxic | Water-dispersible Granules | Solid for Direct Use | [>200µg/bee product,  flufenacet+diflufenican, WG60] (D) | Nontoxic |
| Dimethachlor | Herbicide | Chloroacetanilide | >200 (D) | Nontoxic |  |  |  | No Data |
| Dimethenamid | Herbicide | Amide | >94 (A, D) | Nontoxic |  |  |  | No Data |
| Dimethoate | Insecticide | Organophosphate | 0.19, 0.16,  0.17 (A), 0.1 (D) | Highly Toxic |  |  |  | No Data |
| Dimethomorph | Fungicide | Morpholine | >10, >50 (A),  >102 (D) | Inconclusive |  |  |  | No Data |

| **TGAI** | **Pesticide Type** | **Chemical Class** | **Mined TGAI LD_50_ (µg a.i./bee)** | **TGAI**  **Toxicity Classification** | **TEP Type** | **Super -TEP** | **Mined TEP LD_50_ (µg a.i./bee)** | **TEP Toxicity Classification** |
| --- | --- | --- | --- | --- | --- | --- | --- | --- |
| Dimoxystrobin | Fungicide | Strobin | >100 (D) | Nontoxic | Aqueous Suspension | Liquid Suspensions | >1093  [133g/L+50g/L epoxiconazole] (D) | Nontoxic |
| Diquat | Herbicide | Bipyridylium | 47, 100 (A),  60 (C)  [Geomean 65.6] | Nontoxic | Soluble Concentrate | Liquid Simple Solutions | 60 [SL200] (D) | Nontoxic |
| Dithianon | Fungicide | Unknown | >100 (D) | Nontoxic |  |  |  | No Data |
| Epoxiconazole | Fungicide | Unknown | >100 (D) | Nontoxic |  |  |  | No Data |
| Esfenvalerate | Insecticide | Pyrethroid | 0.0172 (A),  0.06 (C, D) [Geomean 0.032] | Highly Toxic | Emulsifiable Concentrate | Liquid Solutions | 0.07 [50g/L] (C, D) | Highly Toxic |
| Ethofumesate | Herbicide | Unclassified | >50 (A, D),  >100 (D) | Nontoxic |  |  |  | No Data |
| Etofenprox | Insecticide | Pyrethroid | 0.015 (A, D) | Highly Toxic | Emulsifiable Concentrate | Liquid Solutions | 0.33 [1.1ug/bee product, Trebon 30 EC] (D) | Highly Toxic |
| Etoxazole | Miticide | Diphenyl oxazoline | >200 (A, D) | Nontoxic | Aqueous Suspension | Liquid Suspensions | >100  [110g/L,Borneo] (D) | Nontoxic |
| Famoxadone | Fungicide | Oxazole | >97.3 (A),  >100 (D) | Nontoxic | Emulsifiable Concentrate | Liquid Solutions | >200 [100g/L+106.7g  fluzilazole/L] (D) | Nontoxic |
| Famoxadone | Fungicide | Oxazole | >97.3 (A),  >100 (D) | Nontoxic | Water-dispersible Granules | Solid for Direct Use | >200  [22.5%+30% cymoxanil WG] (D) | Nontoxic |
| Fenamidone | Fungicide | Imidazole | 47.1 (A), 74.8 (D) | Nontoxic | Water-dispersible Granules | Solid for Direct Use | >100 [4.44% +  66.7% Fosetyl Al,Vitera Fl] | Nontoxic |
| Fenazaquin | Miticide | Unknown | 8.6, 1.1 (A),  8.18 (D) | Highly Toxic |  |  |  | No Data |
| Fenbuconazole | Fungicide | Azole | >292 (A, D) | Nontoxic |  |  |  | No Data |

| **TGAI** | **Pesticide Type** | **Chemical Class** | **Mined TGAI LD_50_ (µg a.i./bee)** | **TGAI**  **Toxicity Classification** | **TEP Type** | **Super -TEP** | **Mined TEP LD_50_ (µg a.i./bee)** | **TEP Toxicity Classification** |
| --- | --- | --- | --- | --- | --- | --- | --- | --- |
| Fenhexamid | Fungicide | Anilide | >215 (A),  >200 (D) | Nontoxic |  |  |  | No Data |
| Fenoxaprop-P- ethyl | Herbicide | Aryloxyphenoxy propionic acid | >191.4 (A)  >200, >300 (D) | Nontoxic | Emulsion, oil-in- water | Liquid Emulsions | >36.4 [Puma S  69EW] (D) | Nontoxic |
| Fenoxycarb | Insecticide | Other carbamate, JH mimic | >100 (A),  >204 (D) | Nontoxic |  |  |  | No Data |
| Fenpropidin | Fungicide | Unclassified | 46 (D) | Nontoxic | Emulsifiable Concentrate | Liquid Solutions | 55.3 [750g/L, Tern750EC] (D) | Nontoxic |
| Fenpropimorph | Fungicide | Morpholine | >100 (A, D) | Nontoxic | Emulsifiable Concentrate | Liquid Solutions | >79.5 [750g/L,  Corbel] (D) | Nontoxic |
| Fenpyroximate | Miticide | Pyrazole | >479.8 (A) | Nontoxic |  |  |  | No Data |
| Fipronil | Insecticide | Pyrazole | 0.0121,  0.00386,  0.103 (A), 0.00593 (D) | Highly Toxic |  |  |  | No Data |
| Flazasulfuron | Herbicide | Sulfonylurea | >100 (A, D) | Nontoxic | Water-dispersible Granules | Solid for Direct Use | >100 [WG25%] (D) | Nontoxic |
| Flonicamid | Insecticide | Unclassified | >100 (A, D) | Nontoxic | Water-dispersible Granules | Solid for Direct Use | >51.1 [50%WG] (D) | Nontoxic |
| Florasulam | Herbicide | Triazolopyrimidine | >100 (D) | Nontoxic |  |  |  | No Data |
| Fluazifop-P-butyl | Herbicide | Aryloxyphenoxy propionic acid | 63 (A), >200 (D) | Nontoxic | Emulsifiable Concentrate | Liquid Solutions | >100 [125g/L] (D) | Nontoxic |
| Fluazinam | Fungicide | 2,6-Dinitroaniline | >4 (A), >200 (A, D) | Inconclusive |  |  |  | No Data |
| Fludioxonyl | Fungicide | Unclassified | >100 (D) | Nontoxic |  |  |  | No Data |
| Flufenacet | Herbicide | Anilide | >25 (A), >194,  >387 (D) | Nontoxic |  |  |  | No Data |

| **TGAI** | **Pesticide Type** | **Chemical Class** | **Mined TGAI LD_50_ (µg a.i./bee)** | **TGAI**  **Toxicity Classification** | **TEP Type** | **Super -TEP** | **Mined TEP LD_50_ (µg a.i./bee)** | **TEP Toxicity Classification** |
| --- | --- | --- | --- | --- | --- | --- | --- | --- |
| Flumioxazin | Herbicide | N-  phenylphtalamides | >105 (D) | Nontoxic | Wettable Powders | Solid for Direct Use | >200 [Pledge WP50%] (D) | Nontoxic |
| Fluopyram | Fungicide | Amide | >83.2 (A),  >100 (A, D) | Nontoxic | Aqueous Suspension | Liquid Suspensions | [>200µg/bee product, SC500] (D) | Nontoxic |
| Fluoxastrobin | Fungicide | Strobin | >200 (A, D) | Nontoxic | Emulsifiable Concentrate | Liquid Solutions | 29.7 [100g/L] (D) | Nontoxic |
| Flupyrsulfuron methyl | Herbicide | Sulfonylurea | >25 (D) | Nontoxic |  |  |  | No Data |
| Fluquinconazole | Fungicide | Azole | >100 (D) | Nontoxic |  |  |  | No Data |
| Flurochloridone | Herbicide | Unknown | >100 (D) | Nontoxic |  |  |  | No Data |
| Fluroxypyr | Herbicide | Pyridinecarboxylic acid | >25 (A) | Nontoxic | Emulsifiable Concentrate | Liquid Solutions | >180 [259g/L] (D) | Nontoxic |
| Flurtamone | Herbicide | Unclassified | >100 (D) | Nontoxic |  |  |  | No Data |
| Flusilazole | Fungicide | Azole | 165 (D) | Nontoxic |  |  |  | No Data |
| Flutolanil | Fungicide | Anilide | >200 (D) | Nontoxic |  |  |  | No Data |
| Flutriafol | Fungicide | Azole | >10.5, 198  (A), >50 (A, D) | Inconclusive |  |  |  | No Data |
| Folpel | Fungicide | Thiophthalimide | >200 (D) | Nontoxic | Water-dispersible Granules | Solid for Direct Use | >160 [Folpan 80 WDG] (D) | Nontoxic |
| Foramsulfuron | Herbicide | Sulfonylurea | >1.9 (A, D) | Inconclusive | Aqueous Suspension | Liquid Suspensions | >392.2 [22.5g/L+  22.5g/L isoxadifen- ethyl, Equip] | Nontoxic |
| Formetanate | Insecticide | N-Methyl Carbamate | 4.971, 14.27  (A), 1.02 (D) | Highly Toxic |  |  |  | No Data |
| Fosetyl-Al | Fungicide | Organophosphate | >100 (A, D),  >1000 (D) | Nontoxic | Water-dispersible Granules | Solid for Direct Use | >390 [800g/kg,  Aliette], >100 [667g/kg + 44.4 g/k fenamidone, Vitera] (D) | Nontoxic |
| Fosthiazate | Nematicide | Organophosphate | 0.256 (D) | Highly Toxic |  |  |  | No Data |

| **TGAI** | **Pesticide Type** | **Chemical Class** | **Mined TGAI LD_50_ (µg a.i./bee)** | **TGAI**  **Toxicity Classification** | **TEP Type** | **Super -TEP** | **Mined TEP LD_50_ (µg a.i./bee)** | **TEP Toxicity Classification** |
| --- | --- | --- | --- | --- | --- | --- | --- | --- |
| Gamma Cyhalothrin | Insecticide | Pyrethroid | 0.005 (D) | Highly Toxic | Capsule Suspension | Liquid Suspensions | 0.03 [150g/L] (D) | Highly Toxic |
| Glufosinate ammonium | Herbicide | Phosphonoglycine | >100 (A),  >345 (A, D) | Nontoxic |  |  |  | No Data |
| Glyphosate | Herbicide | Phosphonoglycine | >100 (A, D) | Nontoxic | Soluble Concentrate | Liquid Simple Solutions | >100 [360g/L] (D) | Nontoxic |
| Haloxyfop-R | Herbicide | Aryloxyphenoxy propionic acid | >100 (B, D) | Nontoxic | Emulsifiable Concentrate | Liquid Solutions | 96 [EF-1400] (B,D) | Nontoxic |
| Hexythiazox | Miticide | Unclassified | >200 (A, D) | Nontoxic |  |  |  | No Data |
| Hymexazol | Fungicide | Unknown |  | No Data |  |  |  | No Data |
| Imazalil | Fungicide | Azole | 39 (C, D) | Nontoxic |  |  |  | No Data |
| Imazamox | Herbicide | Imidazolinone | >58 (B, C, D),  >25 (D) | Nontoxic | Soluble Concentrate | Liquid Simple Solutions | [>2500µg/bee product, 40 g/L] (B) | Nontoxic |
| Imazaquin | Herbicide | Imidazolinone | >100 (D) | Nontoxic |  |  |  | No Data |
| Imazosulfuron | Fungicide | Sulfonylurea | >100 (B, C) | Nontoxic | Water-dispersible Granules | Solid for Direct Use | >100 [50WG] (B) | Nontoxic |
| Imidacloprid | Insecticide | Neonicotinoid | 0.081 (B, D) | Highly Toxic | Soluble Concentrate | Liquid Simple Solutions | 0.042 [SL200] (B) | Highly Toxic |
| Indoxacarb | Insecticide | Unclassified | 0.18, 0.07 (D)  [Geomean 0.112] | Highly Toxic | Water-dispersible Granules | Solid for Direct Use | 1.34 (60%WG) (D) | Highly Toxic |
| Iodosulfuron | Herbicide | Sulfonylurea | >150 (D) >100 (B) | Nontoxic | Water-dispersible Granules | Solid for Direct Use | >75 (Hussar 20WG) (D) | Nontoxic |
| Ioxynil Octanoate | Herbicide | Hydroxybenzonitrile | >200 (D) | Nontoxic |  |  |  | No Data |
| Ioxynil Phenol | Herbicide | Hydroxybenzonitrile | >100 (D) | Nontoxic |  |  |  | No Data |
| Ipconazole | Fungicide | Azole | >100 (D) | Nontoxic |  |  |  | No Data |
| Iprodione | Fungicide | Dicarboximide | >200 (C, D) | Nontoxic |  |  |  | No Data |

| **TGAI** | **Pesticide Type** | **Chemical Class** | **Mined TGAI LD_50_ (µg a.i./bee)** | **TGAI**  **Toxicity Classification** | **TEP Type** | **Super -TEP** | **Mined TEP LD_50_ (µg a.i./bee)** | **TEP Toxicity Classification** |
| --- | --- | --- | --- | --- | --- | --- | --- | --- |
| Iprovalicarb | Fungicide | Carbamate | >200 (B, C, D) | Nontoxic |  |  |  | No Data |
| Iron sulphate | Herbicide | Inorganic |  | No Data |  |  |  | No Data |
| Isoproturon | Herbicide | Urea | 200 (D) | Nontoxic | Aqueous Suspension | Liquid Suspensions | [>100µg/bee product, 500SC] (B) | Nontoxic |
| Isopyrazam | Fungicide | Pyrazole | >200 (D) | Nontoxic | Emulsifiable Concentrate | Liquid Solutions | 63.64 [125 g/L] (B) | Nontoxic |
| Isoxaben | Herbicide | Benzamide | >100 (B, D) | Nontoxic | Aqueous Suspension | Liquid Suspensions | >47.1 [500SC, EAF-  496] (B) | Nontoxic |
| Isoxaflutole | Herbicide | Oxazole | >100 (B, C, D) | Nontoxic |  |  |  | No Data |
| Kresoxim-methyl | Fungicide | Strobin | >100 (D) | Nontoxic |  |  |  | No Data |
| lambda- Cyhalothrin | Insecticide | Pyrethroid | 0.038 (B, D) | Highly Toxic | Emulsifiable Concentrate | Liquid Solutions | 0.112 [50g/L] (B) | Highly Toxic |
| lambda- Cyhalothrin | Insecticide | Pyrethroid | 0.038 (B, D) | Highly Toxic | Capsule Suspension | Liquid Suspensions | 0.43 [100CS] (B) | Highly Toxic |
| Lenacil | Herbicide | Uracil | >25 (B) | Nontoxic | Wettable Powders | Solid for Direct Use | >100 [800 g/kg] (B) | Nontoxic |
| Linuron | Herbicide | Urea | >1600 (D) | Nontoxic |  |  |  | No Data |
| Lufenuron | Insecticide | Benzoylurea | >200 (B) >8 (D) | Inconclusive |  |  |  | No Data |
| Malathion | Insecticide | Organophosphate | 0.231, 0.64,  1.01, 0.27,  0.22, 0.709  (A) [Geomean 0.429] | Highly Toxic | Emulsion, oil-in- water | Liquid Emulsions | 0.16 [440EW] (D) | Highly Toxic |
| Mancozeb | Fungicide | Dithiocarbamate | 161.7 (B, D) | Nontoxic |  |  |  | No Data |
| Mandipropamid | Fungicide | Amide | >200 (D) | Nontoxic | Aqueous Suspension | Liquid Suspensions | >215 [250 g/L, A12946B] (D) | Nontoxic |

| **TGAI** | **Pesticide Type** | **Chemical Class** | **Mined TGAI LD_50_ (µg a.i./bee)** | **TGAI**  **Toxicity Classification** | **TEP Type** | **Super -TEP** | **Mined TEP LD_50_ (µg a.i./bee)** | **TEP Toxicity Classification** |
| --- | --- | --- | --- | --- | --- | --- | --- | --- |
| Maneb | Fungicide | Dithiocarbamate | >100 (D) | Nontoxic |  |  |  | No Data |
| MCPA | Herbicide | Chlorophenoxy acid | 200 (D) | Nontoxic |  |  |  | No Data |
| MCPB | Herbicide | Chlorophenoxy acid | >100 (D) | Nontoxic |  |  |  | No Data |
| Mecoprop | Herbicide | Chlorophenoxy acid | >100 (D) | Nontoxic |  |  |  | No Data |
| Mecoprop-P | Herbicide | Chlorophenoxy acid | >100 (B, D) | Nontoxic |  |  |  | No Data |
| Mepanipyrim | Fungicide | Anilinopyrimidine | >100 (D) | Nontoxic | Wettable Powders | Solid for Direct Use | >51.1 [50%WP, KIF-3535] (D) | Nontoxic |
| Meptyldinocap | Fungicide | Dinitrophenol |  | No Data | Emulsifiable Concentrate | Liquid Solutions | 84.8 [34.7%, GF1478] (D) | Nontoxic |
| Mesosulfuron | Herbicide | Sulfonylurea | >100 (B) >13 (D) | Nontoxic |  |  |  | No Data |
| Mesotrione | Herbicide | Benzoylcyclohexane dione | >100 (B, D) | Nontoxic | Aqueous Suspension | Liquid Suspensions | 53 [100 g/L SC] (B) | Nontoxic |
| Metalaxyl | Fungicide | Phenylamide | >100 (B) | Nontoxic | Emulsifiable Concentrate | Liquid Solutions | >200 [480g/L] (D) | Nontoxic |
| Metaldehyde | Molluscicide | Aldehyde | >113 (B, D) | Nontoxic |  |  |  | No Data |
| Metamitron | Herbicide | Triazinone | >100 (D) | Nontoxic | Aqueous Suspension | Liquid Suspensions | >200 [Goltix 700SC] (D) | Nontoxic |
| Metazachlor | Herbicide | Chloroacetanilide | >100 (D) | Nontoxic | Aqueous Suspoemulsion | Liquid Dual Character | >100 [500SE] (D) | Nontoxic |
| Metconazole | Fungicide | Azole | >100 (B, D) | Nontoxic |  |  |  | No Data |
| Methiocarb | Insecticide | Methyl Carbamate | 0.23 (D) | Highly Toxic |  |  |  | No Data |
| Methomyl | Insecticide | Carbamate | 0.16 (A, B)  1.29 (D) (Geomean 0.454] | Highly Toxic | Soluble Concentrate | Liquid Simple Solutions | 0.17 (20SL) (B) | Highly Toxic |

| **TGAI** | **Pesticide Type** | **Chemical Class** | **Mined TGAI LD_50_ (µg a.i./bee)** | **TGAI**  **Toxicity Classification** | **TEP Type** | **Super -TEP** | **Mined TEP LD_50_ (µg a.i./bee)** | **TEP Toxicity Classification** |
| --- | --- | --- | --- | --- | --- | --- | --- | --- |
| Methoxyfenozide | Insecticide | Diacylhydrazine | >100 (D) | Nontoxic | Aqueous Suspension | Liquid Suspensions | >50.6 [>200µg/bee product, RH-2485 240SC] (D) | Nontoxic |
| Metiram zinc | Fungicide | Dithiocarbamate | >80 (D) | Nontoxic |  |  |  | No Data |
| Metosulam | Herbicide | Triazolopyrimidine | >100 (B, D) | Nontoxic |  |  |  | No Data |
| Metrafenone | Fungicide | Benzophenone | >100 (D) | Nontoxic | Aqueous Suspension | Liquid Suspensions | >28.8 [>100 µg/bee product, 300SC], >48.9 [>100 µg/bee product, 500SC] (D) | Nontoxic |
| Metribuzin | Herbicide | Triazinone | 200 (D) | Nontoxic | Water-dispersible Granules | Solid for Direct Use | 200 [Metribuzin  70WG], 700  [Mistral 700 W] (D) | Nontoxic |
| Metsulfuron- methyl | Herbicide | Sulfonylurea | >25 (B, D) | Nontoxic | Water-soluble Granules | Solid for Direct Use | >100 [20 SG] (B) | Nontoxic |
| Milbemectin | Insecticide | Macrocyclic Lactone | 0.026 (D) | Highly Toxic | Emulsifiable Concentrate | Liquid Solutions | >1 [100ug/bee  product, 1% EC] (D) | Inconclusive |
| Myclobutanil | Fungicide | Azole |  | No Data | Emulsion, oil-in- water | Liquid Emulsions | 39.6 [200 g/L EW] (B) | Nontoxic |
| Napropamide | Herbicide | Amide |  | No Data | Aqueous Suspension | Liquid Suspensions | >100 [Devrinol 450 SC] (B) | Nontoxic |
| Nicosulfuron | Herbicide | Sulfonylurea | 76 (D) | Nontoxic | Aqueous Suspension | Liquid Suspensions | >5.24 [131µg/bee product, SL-950 4% SC] (D) | Inconclusive |
| Oryzalin | Herbicide | 2,6-Dinitroaniline | 11 (A) | Moderately Toxic | Emulsifiable Concentrate | Liquid Solutions | 40.8 [480 g/L] (D) | Nontoxic |
| Oxadiargyl | Herbicide | Unclassified | >200 (D) | Nontoxic |  |  |  | No Data |
| Oxadiazon | Herbicide | Oxidiazole | >100 (B) >400 (D) | Nontoxic | Emulsifiable Concentrate | Liquid Solutions | >100 [250 g/L] (B) | Nontoxic |
| Oxamyl | Insecticide | Carbamate | 0.47 (D) | Highly Toxic | Soluble Concentrate | Liquid Simple Solutions | 0.23 [10% SL] (D) | Highly Toxic |
| Oxasulfuron | Herbicide | Sulfonylurea | >200 (D) | Nontoxic | Water-dispersible Granules | Solid for Direct Use | >741 [Dynam 75WG] (D) | Nontoxic |

| **TGAI** | **Pesticide Type** | **Chemical Class** | **Mined TGAI LD_50_ (µg a.i./bee)** | **TGAI**  **Toxicity Classification** | **TEP Type** | **Super -TEP** | **Mined TEP LD_50_ (µg a.i./bee)** | **TEP Toxicity Classification** |
| --- | --- | --- | --- | --- | --- | --- | --- | --- |
| Oxathiapiprolin | Fungicide | Piperidinyl thiazole isoxazoline | >100 (B) | Nontoxic | Oil-Based Suspension | Liquid Suspensions | >100 [100 g/L] (B) | Nontoxic |
| Oxyfluorfen | Herbicide | Diphenyl Ether | >100 (B, D) | Nontoxic |  |  |  | No Data |
| Penconazole | Fungicide | Azole | >100 | Nontoxic | Emulsifiable Concentrate | Liquid Solutions | >0.30 [>30 µg/bee product, Topas 10 EC] (B) | Inconclusive |
| Pencycuron | Fungicide | Urea | 100 (B, D) | Nontoxic |  |  |  | No Data |
| Pendimethalin | Herbicide | 2,6-Dinitroaniline | >100 (B, D) | Nontoxic | Aqueous Suspension | Liquid Suspensions | >200 [400 SC] (B) | Nontoxic |
| Pendimethalin | Herbicide | 2,6-Dinitroaniline | >100 (B, D) | Nontoxic | Capsule Suspension | Liquid Suspensions | [259.1µg/bee product, 455 g/L) (B) | Nontoxic |
| Penoxsulam | Herbicide | Triazolopyrimidine | >100 (D) | Nontoxic | Oil-Based Suspension | Liquid Suspensions | [>100µg/bee product, 20.4 g/L] (B) | Nontoxic |
| Penthiopyrad | Fungicide | Amide | >500 (B) | Nontoxic | Emulsifiable Concentrate | Liquid Solutions | 23.5 [200 g/L EC] (B) | Nontoxic |
| Penthiopyrad | Fungicide | Amide | >500 (B) | Nontoxic | Aqueous Suspension | Liquid Suspensions | >100 [200 g/L] (B) | Nontoxic |
| Pethoxamid | Herbicide | Amide | 200 (D) | Nontoxic |  |  |  | No Data |
| Phenmedipham | Herbicide | Bis-Carbamate | 50 (D) | Nontoxic |  |  |  | No Data |
| Phosmet | Insecticide | Organophosphate | 0.22 (B, D) | Highly Toxic |  |  |  | No Data |
| Picloram | Herbicide | Pyridinecarboxylic acid | >100 (D) | Nontoxic |  |  |  | No Data |
| Picolinafen | Herbicide | Pyridinecarboxylic acid | >200 (B, D) | Nontoxic | Water-dispersible Granules | Solid for Direct Use | >200 [Picosolo 750 g/kg WG] (B, D) | Nontoxic |
| Picoxystrobin | Fungicide | Strobin | >200 (B, D) | Nontoxic | Aqueous Suspension | Liquid Suspensions | >200 [250g/L] (B, D) | Nontoxic |
| Pinoxaden | Herbicide | Unclassified | >100 (B, D) | Nontoxic | Emulsifiable Concentrate | Liquid Solutions | 8.17 (B) | Moderately Toxic |

| **TGAI** | **Pesticide Type** | **Chemical Class** | **Mined TGAI LD_50_ (µg a.i./bee)** | **TGAI**  **Toxicity Classification** | **TEP Type** | **Super -TEP** | **Mined TEP LD_50_ (µg a.i./bee)** | **TEP Toxicity Classification** |
| --- | --- | --- | --- | --- | --- | --- | --- | --- |
| Pirimicarb | Insecticide | Carbamate | 53.1 (B, D) | Nontoxic | Water-dispersible Granules | Solid for Direct Use | 51.1 [500 WG] (B) | Nontoxic |
| Pirimiphos-methyl | Insecticide | Organophosphate |  | No Data |  |  |  | No Data |
| Potassium phosphonate | Fungicide | Inorganic | >207 (B)  >71.87 (D) | Nontoxic |  |  |  | No Data |
| Prochloraz | Fungicide | Azole | 141.28 (B, D) | Nontoxic | Emulsion, oil-in- water | Liquid Emulsions | 46.7 [BAS 590 00 F, 450 g/L EW] (B, D) | Nontoxic |
| Propamocarb | Fungicide | Carbamate | >100 (B, D) | Nontoxic |  |  |  | No Data |
| Propaquizafop | Herbicide | Aryloxyphenoxy propionic acid | >200 (D) | Nontoxic | Emulsifiable Concentrate | Liquid Solutions | >18.9 [>189 µg product/bee , 100 g/L] (D) | Nontoxic |
| Propiconazole | Fungicide | Azole | >100 (D), >25 | Nontoxic |  |  |  | No Data |
| Propineb | Fungicide | Dithiocarbamate | >164 (D) | Nontoxic |  |  |  | No Data |
| Propoxycarbazone | Herbicide | Triazolone | >200 (D) | Nontoxic | Water-dispersible Granules | Solid for Direct Use | >200 (70% WG) (D) | Nontoxic |
| Propyzamide | Herbicide | Amide | >136 (D) | Nontoxic |  |  |  | No Data |
| Proquinazid | Fungicide | Quinazolinone | >197 (D) | Nontoxic | Emulsifiable Concentrate | Liquid Solutions | >100 [200 g/L] (D) | Nontoxic |
| Prosulfocarb | Herbicide | Thiocarbamate | >80 (B, D) | Nontoxic | Emulsifiable Concentrate | Liquid Solutions | >79.3 [80EC, SF245] (B, D) | Nontoxic |
| Prosulfuron | Herbicide | Sulfonylurea | >100 (B) | Nontoxic | Water-dispersible Granules | Solid for Direct Use | >100 (750 g/kg  WG) (B), >105 (63WG) (D) | Nontoxic |
| Prothioconazole | Fungicide | Azole | >200 (D) | Nontoxic | Emulsifiable Concentrate | Liquid Solutions | >200 [250 g/L] (D) | Nontoxic |
| Pymetrozine | Insecticide | Pyridine | >200 (B, D) | Nontoxic | Water-dispersible Granules | Solid for Direct Use | [100 µg product/bee, 500 g/kg] (B) | Nontoxic |
| Pyraclostrobin | Fungicide | Strobin | >100 (D) | Nontoxic | Emulsifiable Concentrate | Liquid Solutions | >100 [250g/L] (D) | Nontoxic |

| **TGAI** | **Pesticide Type** | **Chemical Class** | **Mined TGAI LD_50_ (µg a.i./bee)** | **TGAI**  **Toxicity Classification** | **TEP Type** | **Super -TEP** | **Mined TEP LD_50_ (µg a.i./bee)** | **TEP Toxicity Classification** |
| --- | --- | --- | --- | --- | --- | --- | --- | --- |
| Pyraflufen-ethyl | Herbicide | Phenylpyrazole | >100 (B, D) | Nontoxic | Emulsifiable Concentrate | Liquid Solutions | 5.79 [2.5% EC] (B) | Moderately Toxic |
| Pyrethrin | Insecticide | Pyrethroid | 0.013 (B) 0.13-0.29,  0.022 (D) [Geomean 0.057] | Highly Toxic | Emulsifiable Concentrate | Liquid Solutions | 0.4 [18.61 g/L] (B) | Highly Toxic |
| Pyridaben | Insecticide | Unclassified | 0.024 (A, B), 0.1163 (A) [Geomean 0.0528] | Highly Toxic | Aqueous Suspension | Liquid Suspensions | 0.691 [SC] (B) | Highly Toxic |
| Pyridaben | Insecticide | Unclassified | 0.024 (A, B), 0.1163 (A) [Geomean 0.0528] | Highly Toxic | Wettable Powders | Solid for Direct Use | 3.68 [750 g/kg] (B) | Moderately Toxic |
| Pyridate | Herbicide | Phenylpyridazine | >100 (B, D) | Nontoxic | Wettable Powders | Solid for Direct Use | >100 [450 g/kg] (B) | Nontoxic |
| Pyrimethanil | Fungicide | Pyrimidine | >100 (D) | Nontoxic | Aqueous Suspension | Liquid Suspensions | >200 [400g/L] (D) | Nontoxic |
| Pyriofenone | Fungicide | Aryl phenyl ketone | >100 (B, D) | Nontoxic |  |  |  | No Data |
| Pyriproxyfen | Insecticide | Unclassified | >100 (D) | Nontoxic | Emulsifiable Concentrate | Liquid Solutions | >100 [10EC] (B) | Nontoxic |
| Pyroxsulam | Herbicide | Triazolopyrimidine | >100 (B, D) | Nontoxic | Water-dispersible Granules | Solid for Direct Use | >104 [75WG] (B) | Nontoxic |
| Quinmerac | Herbicide | Quinolinecarboxylic acid | >100 (B, D) | Nontoxic |  |  |  | No Data |
| Quinoclamine | Herbicide | Unclassified |  | No Data |  |  |  | No Data |
| Quinoxyfen | Fungicide | Quinoline | >100 (B, D) | Nontoxic | Aqueous Suspension | Liquid Suspensions | >100 [500 SC] (B) | Nontoxic |

| **TGAI** | **Pesticide Type** | **Chemical Class** | **Mined TGAI LD_50_ (µg a.i./bee)** | **TGAI**  **Toxicity Classification** | **TEP Type** | **Super -TEP** | **Mined TEP LD_50_ (µg a.i./bee)** | **TEP Toxicity Classification** |
| --- | --- | --- | --- | --- | --- | --- | --- | --- |
| Quizalofop-P | Herbicide | Aryloxyphenoxy propionic acid |  | No Data |  |  |  | No Data |
| Quizalofop-P ethyl | Herbicide | Aryloxyphenoxy propionic acid | >100 (B, D) | Nontoxic | Emulsifiable Concentrate | Liquid Solutions | >25 [50g/L] (B, D) | Nontoxic |
| Quizalofop-P tefuryl | Herbicide | Aryloxyphenoxy propionic acid | >100 (B, D) | Nontoxic | Emulsifiable Concentrate | Liquid Solutions | >40 [40g/L] (B, D) | Nontoxic |
| Rimsulfuron | Herbicide | Sulfonylurea | >100 (A) | Nontoxic | Water-dispersible Granules | Solid for Direct Use | 27.9 [25 WG +surfactant Excell] (B, D) | Nontoxic |
| Sedaxane | Fungicide | Pyrazole | > 100 (B, D) | Nontoxic | Suspension for Seeds | Liquid Suspensions | >42.8 [>100µg product/bee, 500g/l FS] (B) | Nontoxic |
| Silthiofam | Fungicide | Unclassified |  | No Data |  |  |  | No Data |
| Sintofen | Plant Growth Regulator | Unclassified |  | No Data | Soluble Concentrate | Liquid Simple Solutions | > 100 [100 g/l SL] (B, D) | Nontoxic |
| S-metolachlor | Herbicide | Chloroacetanilide | >100 (A) >  200 (C, D) | Nontoxic | Emulsifiable Concentrate | Liquid Solutions | >200 [960 g/L, A-9396 B] (D) | Nontoxic |
| Spinosad | Insecticide | Macrocyclic Lactone | 0.0036 (C, D) | Highly Toxic | Aqueous Suspension | Liquid Suspensions | 0.050 [480 g/L] (C) | Highly Toxic |
| Spirodiclofen | Insecticide | tetronic acid | >200 (B, D) | Nontoxic | Aqueous Suspension | Liquid Suspensions | >100 [240 g/L, BAJ 2740 240 SC] (B, D) | Nontoxic |
| Spirotetramat | Insecticide | Tetramic acid | >100 (B, D) | Nontoxic | Oil-Based Suspension | Liquid Suspensions | 162 [150 g/L] (B) | Nontoxic |
| Spiroxamine | Fungicide | Morpholine | 4.2 (A, B, D) | Moderately Toxic | Emulsifiable Concentrate | Liquid Solutions | 30 [500 g/L, KWG4168] (B, D); >200 [500 g/L, KWG4168] (B, D) | Nontoxic |
| Sulcotrione | Herbicide | Benzoylcyclohexane dione | 200 (B), >200 (D) | Nontoxic | Aqueous Suspension | Liquid Suspensions | [>763µg product/bee, 300 g/L] (B) | Nontoxic |
| Sulfosulfuron | Herbicide | Sulfonylurea | >25 (A, B, D) | Nontoxic | Water-dispersible Granules | Solid for Direct Use | >100 [800 g/kg WG] (B) | Nontoxic |

| **TGAI** | **Pesticide Type** | **Chemical Class** | **Mined TGAI LD_50_ (µg a.i./bee)** | **TGAI**  **Toxicity Classification** | **TEP Type** | **Super -TEP** | **Mined TEP LD_50_ (µg a.i./bee)** | **TEP Toxicity Classification** |
| --- | --- | --- | --- | --- | --- | --- | --- | --- |
| Sulfur | Fungicide | Inorganic | > 100 dust (B) | Nontoxic | Water-dispersible Granules | Solid for Direct Use | > 100 [80% WG] (B) | Nontoxic |
| Tau-fluvalinate | Miticide | Pyrethroid | 12 (A, B, D) | Nontoxic | Emulsion, oil-in- water | Liquid Emulsions | 128 [Mavrik 2F, 240 g/l EW] (B, D) | Nontoxic |
| Tebuconazole | Fungicide | Azole | >200 (B, D) | Nontoxic | Emulsion, oil-in- water | Liquid Emulsions | 143 (48h), 97 (72h) [Folicur EW 250] (B) | Nontoxic |
| Tebufenozide | Insecticide | Diacylhydrazine (molting hormone agonist) | >234 (A, B, D) | Nontoxic |  |  |  | No Data |
| Tebufenpyrad | Miticide | Pyrazole | 6.7(B, D) | Moderately Toxic | Wettable Powders | Solid for Direct Use | 71 [200 g/kg]  (B,D); 75.9 [200  g/kg] (B, D) | Nontoxic |
| Teflubenzuron | Insecticide | Benzoylurea | 100 (B),  >1000 (D) | Nontoxic | Aqueous Suspension | Liquid Suspensions | 100 [150 g/l SC] (B) | Nontoxic |
| Tefluthrin | Insecticide | Pyrethroid | 0.28 (B, D) | Highly Toxic |  |  |  | No Data |
| Tembotrione | Herbicide | Benzoylcyclohexane dione | > 100 (A, B, D) | Nontoxic | Oil-Based Suspension | Liquid Suspensions | >17 [44 g/L] (B) | Nontoxic |
| Tepraloxydim | Herbicide | Cyclohexenone derivative | > 200 (C, D) | Nontoxic | Emulsifiable Concentrate | Liquid Solutions | >200 [BAS 620 00 H (0.5L) + Dash HC  (2L) | Nontoxic |
| Tetraconazole | Fungicide | Azole | 63 (B, D) | Nontoxic | Microemulsion | Liquid Emulsions | 27.2 [40 g/L] (B) | Nontoxic |
| Thiabendazole | Fungicide | Benzimidazole | > 34 (B, D), | Nontoxic | Aqueous Suspension | Liquid Suspensions | >200 [Tecto 500SC, 500 g/L] (B, D) | Nontoxic |
| Thiacloprid | Insecticide | Neonicotinoid | 38.82 (C, D),  43.59 (A),  37.87 (A) [Geomean 40.02] | Nontoxic | Aqueous Suspension | Liquid Suspensions | 51.6 [480 g/L, YRC 2894] (C, D) | Nontoxic |

| **TGAI** | **Pesticide Type** | **Chemical Class** | **Mined TGAI LD_50_ (µg a.i./bee)** | **TGAI**  **Toxicity Classification** | **TEP Type** | **Super -TEP** | **Mined TEP LD_50_ (µg a.i./bee)** | **TEP Toxicity Classification** |
| --- | --- | --- | --- | --- | --- | --- | --- | --- |
| Thiamethoxam | Insecticide | Neonicotinoid | 0.024 (B, D) | Highly Toxic | Others | Liquid Suspensions | 13.26 g a.s. [Dust from formulation A9700B (dust contained 7.24 % thiamethoxam)] (B) | Nontoxic |
| Thiencarbazone- methyl | Herbicide | Triazolone | >200 (A, B),  >199 (D) | Nontoxic | Aqueous Suspension | Liquid Suspensions | >50.5 [450 g/L, PP DAR SC450] (B, D) | Nontoxic |
| Thifensulfuron- methyl | Herbicide | Sulfonylurea | >98.16 (A); >  7.1 (C, D) | Inconclusive |  |  |  | No Data |
| Thiophanate- methyl | Fungicide | Benzimidazole precursor | >100 (A,C, D) | Nontoxic |  |  |  | No Data |
| Thiram | Fungicide | Dithiocarbamate | 74 (A), >100 (C, D) | Nontoxic |  |  |  | No Data |
| Tolclofos-methyl | Fungicide | Unclassified | >100 (B, D ) | Nontoxic |  |  |  | No Data |
| Tralkoxydim | Herbicide | Cyclohexenone derivative | >100 (B, D) | Nontoxic | Aqueous Suspension | Liquid Suspensions | >100 [242 g/L] (B) | Nontoxic |
| Triadimenol | Fungicide | Azole | >200 (B) | Nontoxic | Emulsifiable Concentrate | Liquid Solutions | >200 [250 g/L] (B) | Nontoxic |
| Triadimenol | Fungicide | Azole | >200 (B) | Nontoxic | Suspension for Seeds | Liquid Suspensions | >2232 [94 g/L FS] (B) | Nontoxic |
| Triallate | Herbicide | Thiocarbamate | >25 (A);  >1000 (A) | Nontoxic |  |  |  | No Data |
| Triasulfuron | Herbicide | Sulfonylurea | >100 (B, D) | Nontoxic | Water-dispersible Granules | Solid for Direct Use | >203 [200 g/kg] (B) | Nontoxic |
| Tribenuron | Herbicide | Sulfonylurea | >98.4 (B, D);  100 (A) | Nontoxic | Water-dispersible Granules | Solid for Direct Use | >200 [75 g/kg] (B, D) | Nontoxic |
| Triclopyr | Herbicide | Pyridine | >100 (B,D) | Nontoxic | Emulsifiable Concentrate | Liquid Solutions | >100 [480 g/L] (B,D) | Nontoxic |
| Trifloxystrobin | Fungicide | Strobin | >200 (A, C, D) | Nontoxic | Emulsifiable Concentrate | Liquid Solutions | >100 [125 g/L,  Twist] (C, D) | Nontoxic |

| **TGAI** | **Pesticide Type** | **Chemical Class** | **Mined TGAI LD_50_ (µg a.i./bee)** | **TGAI**  **Toxicity Classification** | **TEP Type** | **Super -TEP** | **Mined TEP LD_50_ (µg a.i./bee)** | **TEP Toxicity Classification** |
| --- | --- | --- | --- | --- | --- | --- | --- | --- |
| Trifloxystrobin | Fungicide | Strobin | >200 (A, C, D) | Nontoxic | Water-dispersible Granules | Solid for Direct Use | >200 [500 g/kg, Consist] (C, D) | Nontoxic |
| Triflusulfuron | Herbicide | Sulfonylurea | > 100 (B, D);  >25 (A) | Nontoxic |  |  |  | No Data |
| Trinexapac | Plant Growth Regulator | Unclassified | 47(A); >200 (D) | Nontoxic | Emulsifiable Concentrate | Liquid Solutions | 69.6 [250 g/l EC] (B, D) | Nontoxic |
| Triticonazole | Fungicide | Azole | >100 (B, D);  >24 (A) | Nontoxic |  |  |  | No Data |
| Tritosulfuron | Herbicide | Sulfonylurea | 200 (C, D) | Nontoxic | Water-dispersible Granules | Solid for Direct Use | >100 [714 g/kg WG+adjuvant] (C, D) | Nontoxic |
| Valifenalate | Fungicide | Amino acid | > 100 (B, D) | Nontoxic | Water-dispersible Granules | Solid for Direct Use | >184  [6%valifenalate+48  %folpet WG] (B) | Nontoxic |
| Zeta-cypermethrin | Insecticide | Pyrethroid | 0.023; 0.088;  0.13 (A) [Geomean 0.064] | Highly Toxic | Emulsion, oil-in- water | Liquid Emulsions | 0.002  [0.021µg/bee product, 100g/L] (B, D | Highly Toxic |
| Ziram | Fungicide | Dithiocarbamate | 46.6; 100 (A);  >100 (C, D) | Nontoxic |  |  |  | No Data |
| Zoxamide | Fungicide | Benzamide | >100 (A, C, D) | Nontoxic | Wettable Powders | Solid for Direct Use | >200 [80WP, 7-8% zoxamide+67-70% mancozeb] (C, D) | Nontoxic |
| Zoxamide | Fungicide | Benzamide | >100 (A, C, D) | Nontoxic | Water-dispersible Granules | Solid for Direct Use | >200 [75WG, 8.3% Zoxamide +66.7% mancozeb] (C, D) | Nontoxic |
| Zoxamide | Fungicide | Benzamide | >100 (A, C, D) | Nontoxic | Aqueous Suspension | Liquid Suspensions | >200 [240 g/L] (D) | Nontoxic |

Supplemental Table 2. List of all Technical Grade Active Ingredients (TGAI) in alphabetical order, obtained from four publicly available databases to mine the honey bee oral acute endpoint (Lethal median dose or LD_50_, expressed as µg a.i./bee). The four databases were the US Environmental Protection Agency (A), the European Food Safety Authority (B), the European Commission Pesticides Database (C), and the National Institute for Agricultural Research of France (D). The pesticide type, chemical class, type of Typical End-use Product (TEP), Super-TEP category (based on a broader description of the physical characteristics of the formulated product), and toxicity classification for each TGAI are identified.

| **TGAI** | **Pesticide Type** | **Chemical Class** | **Mined TGAI LD_50_ (µg a.i./bee)** | **TGAI**  **Toxicity Classification** | **TEP Type** | **Super -TEP** | **Mined TEP LD_50_ (µg a.i./bee)** | **TEP Toxicity Classification** |
| --- | --- | --- | --- | --- | --- | --- | --- | --- |
| Abamectin | Insecticide | Avermectins |  | No Data |  |  |  | No Data |
| Acequinocyl | Miticide | Quinoline | >100 (D),  >315(A) | Nontoxic | Aqueous Suspension | Liquid Suspensions | >48.5 Kanemite] (D) | Nontoxic |
| Acetamiprid | Insecticide | Neonicotinoid | 14.53 (A, C, D) | Nontoxic | Concentrate | Liquid Solutions | 8.85 [20%C] (C) | Moderately Toxic |
| Acibenzolar-S-  methyl (benzothiadiazole) | Fungicide | Benzothiadiazole | >128 (A, C, D) | Nontoxic | Water-dispersible Granules | Solid for Direct Use | >199 [Bion 50WG] (D) | Nontoxic |
| Aclonifen | Herbicide | Diphenyl ether | >107 (D) | Nontoxic | Aqueous Suspension | Liquid Suspensions | [>236µg/bee product, Bnadur 600SC] (D) | Nontoxic |
| Acrinathrin | Miticide | Pyrethroid | 0.077 (D) | Highly Toxic |  |  |  | No Data |
| Alphamethrin (alpha cypermethrin) | Insecticide | Pyrethroid | 0.059 (C, D) | Highly Toxic | Emulsifiable Concentrate | Liquid Solutions | 0.13 [100EC] (C, D) | Highly Toxic |
| Ametoctradin | Fungicide | Triazolopyrimidine | >111.5 (D) | Nontoxic | Flowable (liquid) | Liquid  Solutions | >67.6  [BAS65100F] (D) | Nontoxic |
| Amitrole (aminotriazole) | Herbicide | Triazole | >152 (C),  >200 (A) | Nontoxic |  |  |  | No Data |
| Azoxystrobin | Fungicide | Strobin | >25 (D) | Nontoxic | Water-dispersible Granules | Solid for Direct Use | >200  [Amistar500] (D) | Nontoxic |
| Azoxystrobin | Fungicide | Strobin | >25 (D) | Nontoxic | Aqueous Suspension | Liquid Suspensions | >200  [Amistar250] (D) | Nontoxic |
| Beflubutamid | Herbicide | Amide | >200 (C, D) | Nontoxic |  |  |  | No Data |
| Benalaxyl | Fungicide | Xylylalanine | >100 (C) | Nontoxic | Wettable Powders | Solid for Direct Use | >250 [Galben M] (C ) | Nontoxic |
| Benfluralin | Herbicide | 2,6-Dinitroaniline | >110.7 (D) | Nontoxic | Emulsifiable Concentrate | Liquid Solutions | >31.25 [182g/L,  Bonalan] (D) | Nontoxic |
| Benoxacor | Herbicide safener | Unknown | >100 (D) | Nontoxic |  |  |  | No Data |
| Bentazone | Herbicide | Unclassified | >200 (C, D) | Nontoxic |  |  |  | No Data |
| Benthiavalicarb- Isopropyl | Fungicide | Carbamate | >100 (D) | Nontoxic | Water-dispersible Granules | Solid for Direct Use | >140 [WG1.75% + 70% mancozeb] (D) | Nontoxic |
| Betacyfluthrin | Insecticide | Pyrethroid | 0.05 (C, D) | Highly Toxic |  |  |  | No Data |

| **TGAI** | **Pesticide Type** | **Chemical Class** | **Mined TGAI LD_50_ (µg a.i./bee)** | **TGAI**  **Toxicity Classification** | **TEP Type** | **Super -TEP** | **Mined TEP LD_50_ (µg a.i./bee)** | **TEP Toxicity Classification** |
| --- | --- | --- | --- | --- | --- | --- | --- | --- |
| Bifenazate | Miticide | Hydrazine carboxylate | >110 (D) | Nontoxic | Aqueous Suspension | Liquid Suspensions | >110 [480g/L] (D) | Nontoxic |
| Bifenox | Herbicide | Diphenyl ether | >200 (D) | Nontoxic |  |  |  | No Data |
| Bifenthrin | Insecticide | Pyrethroid | 0.1 (D) | Highly Toxic | Aqueous Suspension | Liquid Suspensions | 0.01 [Talstar8SC] (D) | Highly Toxic |
| Boscalid | Fungicide | Carboxamide | >166 (A, C, D) | Nontoxic | Water-dispersible Granules | Solid for Direct Use | >100 [CantusWG  50%] (D) | Nontoxic |
| Bromoxynil octanoate | Herbicide | Hydroxybenzonitrile | >119.8 (D) | Nontoxic |  |  |  | No Data |
| Bromoxynil phenol | Herbicide | Hydroxybenzonitrile | 5 (D) | Moderately Toxic |  |  |  | No Data |
| Bromuconazole | Fungicide | Azole | >100 (D) | Nontoxic |  |  |  | No Data |
| Bupirimate | Fungicide | Pyrimidine | >200 (D) | Nontoxic |  |  |  | No Data |
| Captan | Fungicide | Thiophthalimide | >2000 (A),>100 (D) | Nontoxic | Water-dispersible Granules | Solid for Direct Use | >169.3 [Merpan 80WDG] (D) | Nontoxic |
| Carbetamide | Herbicide | Amide |  | No Data | Water-dispersible Granules | Solid for Direct Use | >63.22 [WG60%] (D) | Nontoxic |
| Carboxin | Fungicide | Carboxamide | >181.29 (A),  >100 (D) | Nontoxic |  |  |  | No Data |
| Carfentrazone ethyl | Herbicide | Triazolone |  | No Data | Water-dispersible Granules | Solid for Direct Use | >200 [Aurora WG50%] (D) | Nontoxic |
| Chlorantraniliprole | Insecticide | Anthranilic diamide | >104.1 (D) | Nontoxic | Water-dispersible Granules | Solid for Direct Use | >119.19 [DPX- E2Y45 35WG] (D) | Nontoxic |
| Chlorantraniliprole | Insecticide | Anthranilic diamide | >104.1 (D) | Nontoxic | Aqueous Suspension | Liquid Suspensions | >114.1 [DPX- E2Y45 20SC] (D) | Nontoxic |
| Chloridazone | Herbicide | Pyridazinone | >200 (D) | Nontoxic | Water-dispersible Granules | Solid for Direct Use | [>159µg/bee  product, Pyramin WG] (D) | Nontoxic |
| Chlorothalonil | Fungicide | Substituted  Benzene | >40 (D) | Nontoxic |  |  |  | No Data |
| Chlorotoluron | Herbicide | Urea |  | No Data | Aqueous Suspension | Liquid Suspensions | >105.8 [700g/L] (D) | Nontoxic |
| Chlorpyriphos | Insecticide | Organophosphate | 0.209, 0.302,  0.066, 0.051,  0.11 (A) | Highly Toxic |  |  |  | No Data |
| Chlorpyriphos- ethyl | Insecticide | Organophosphate | 0.25 (D) | Highly Toxic |  |  |  | No Data |

| **TGAI** | **Pesticide Type** | **Chemical Class** | **Mined TGAI LD_50_ (µg a.i./bee)** | **TGAI**  **Toxicity Classification** | **TEP Type** | **Super -TEP** | **Mined TEP LD_50_ (µg a.i./bee)** | **TEP Toxicity Classification** |
| --- | --- | --- | --- | --- | --- | --- | --- | --- |
| Chlorpyriphos- methyl | Insecticide | Organophosphate | 0.11 (D) | Highly Toxic | Emulsifiable Concentrate | Liquid Solutions | 0.18 [EC 225 g/l] (D) | Highly Toxic |
| Clodinafop- propargyl | Herbicide | Aryloxyphenoxy propionic acid | >100 (A),  >93.7 (D) | Nontoxic | Emulsifiable Concentrate | Liquid Solutions | 17.8 [Topik 100 EC] (D) | Nontoxic |
| Clomazone | Herbicide | Unclassified | >85.29 (A, D) | Nontoxic |  |  |  | No Data |
| Clopyralid | Herbicide | Pyridenecarboxylic acid | >100 (A, D) | Nontoxic |  |  |  | No Data |
| Clothianidin | Insecticide | Neonicotinoid | 0.00368 (A),  0.00379,  0.0025 (C, D) | Highly Toxic |  |  |  | No Data |
| Cyazofamid | Fungicide | Azole | >151.7 (A, D) | Nontoxic | Aqueous Suspension | Liquid Suspensions | [>115.7µg/bee product, 400g/L, Ranman] (D) | Nontoxic |
| Cyfluthrin | Insecticide | Pyrethroid |  | No Data | Emulsifiable Concentrate | Liquid Solutions | 0.051 [Baytrhoid EC50] (D) | Highly Toxic |
| Cyhalofop-butyl | Herbicide | Aryloxyphenoxy propionic acid | >100 (A, D) | Nontoxic | Emulsifiable Concentrate | Liquid Solutions | >40 [Clincher EC200] (D) | Nontoxic |
| Cymoxanil | Fungicide | Cyanoacetamide oxime | >85.29 (D) | Nontoxic | Water-dispersible Granules | Solid for Direct Use | 55.5 [with Famoxadone, 52.5WG] (D) | Nontoxic |
| Cypermethrin | Insecticide | Pyrethroid | 0.56, 0.11,  0.172 (A),  0.035 (C, D)  [Geomean 0.1387 | Highly Toxic | Emulsifiable Concentrate | Liquid Solutions | 3.2 [100 EC] (C,D), 0.031 [400 EC] (D) | Highly Toxic |
| Cyproconazole | Fungicide | Azole | >1000 (A),  >100 (D) | Nontoxic | Soluble Concentrate | Liquid Simple Solutions | >1000  [Alto100SL, A- 9898A] (D) | Nontoxic |
| Cyprodinil | Fungicide | Pyrimidine |  | No Data | Water-dispersible Granules | Solid for Direct Use | >150 [Unix 75 WG], >250  [Chorus 50WG] (D) | Nontoxic |
| Deltamethrin | Insecticide | Pyrethroid | 0.186 (A),  0.079 (C, D) [Geomean 0.121] | Highly Toxic | Emulsifiable Concentrate | Liquid Solutions | 0.280 [Decis 25EC] (C, D) | Highly Toxic |
| Desmedipham | Herbicide | Carbamate | >50 (A, D) | Nontoxic | Emulsifiable Concentrate | Liquid Solutions | >48.6 [Betanal AM11] (D) | Nontoxic |

| **TGAI** | **Pesticide Type** | **Chemical Class** | **Mined TGAI LD_50_ (µg a.i./bee)** | **TGAI**  **Toxicity Classification** | **TEP Type** | **Super -TEP** | **Mined TEP LD_50_ (µg a.i./bee)** | **TEP Toxicity Classification** |
| --- | --- | --- | --- | --- | --- | --- | --- | --- |
| Dicamba | Herbicide | Benzoid Acid | 3.6 (A), >100 (D) | Moderately Toxic | Soluble Concentrate | Liquid Simple Solutions | >100 [Banvel 480SL] (D) | Nontoxic |
| Dichlorprop P | Herbicide | Chlorophenoxy acid | >200 (D) | Nontoxic |  |  |  | No Data |
| Diclofop methyl | Herbicide | Aryloxyphenoxy propionic acid | >131 (D) | Nontoxic |  |  |  | No Data |
| Diethofencarb | Fungicide | Carbamate | >100 (D) | Nontoxic | Wettable Powders | Solid for Direct Use | >100 [Powmyl 25WP] (D) | Nontoxic |
| Difenoconazole | Fungicide | Azole | >177 (A, D) | Nontoxic |  |  |  | No Data |
| Diflubenzuron | Insecticide | Benzoylurea | >30 (D) | Nontoxic |  |  |  | No Data |
| Diflufenican | Herbicide | Carboxamide | >112.3 (D) | Nontoxic | Water-dispersible Granules | Solid for Direct Use | [>198µg product/bee, with flufenacet, WG 60] (D) | Nontoxic |
| Dimethachlor | Herbicide | Chloroacetanilide | >300 (D) | Nontoxic |  |  |  | No Data |
| Dimethenamid | Herbicide | Amide | >1000 (A, D) | Nontoxic |  |  |  | No Data |
| Dimethoate | Insecticide | Organophosphate | 1.93, 0.083,  0.0561.67,  1.69, <0.2,  0.3, 0.26, 0.1 | Highly Toxic |  |  |  | No Data |
| Dimethomorph | Fungicide | Morpholine | >32.4 (D) | Nontoxic |  |  |  | No Data |
| Dimoxystrobin | Fungicide | Strobin | >79.4 (D) | Nontoxic | Aqueous Suspension | Liquid Suspensions | >882 [133g/L +50g/L epoxiconazole] (D) | Nontoxic |
| Diquat | Herbicide | Bipyridylium | 13 (C) | Nontoxic | Soluble Concentrate | Liquid Simple Solutions | 13 [SL200] (D) | Nontoxic |
| Dithianon | Fungicide | Unknown | >25.4 (D) | Nontoxic |  |  |  | No Data |
| Epoxiconazole | Fungicide | Unknown | >83 (D) | Nontoxic |  |  |  | No Data |
| Esfenvalerate | Insecticide | Pyrethroid |  | No Data | Emulsifiable Concentrate | Liquid Solutions | 0.21, 0.8 [50EC] (C, D) | Highly Toxic |
| Ethofumesate | Herbicide | Unclassified | >50, >100 (D) | Nontoxic | Aqueous Suspension | Liquid Suspensions | >100 [Tramat 500 /Notron 50 SC] (D) | Nontoxic |
| Etofenprox | Insecticide | Pyrethroid | 0.024 (D) | Highly Toxic | Emulsifiable Concentrate | Liquid Solutions | 0.38 [1.27 µg product/bee,  30% EC] (D) | Highly Toxic |

| **TGAI** | **Pesticide Type** | **Chemical Class** | **Mined TGAI LD_50_ (µg a.i./bee)** | **TGAI**  **Toxicity Classification** | **TEP Type** | **Super -TEP** | **Mined TEP LD_50_ (µg a.i./bee)** | **TEP Toxicity Classification** |
| --- | --- | --- | --- | --- | --- | --- | --- | --- |
| Etoxazole | Miticide | Diphenyl oxazoline | >200 (A, D) | Nontoxic | Aqueous Suspension | Liquid Suspensions | >100 [Borneo 110SC] (D) | Nontoxic |
| Famoxadone | Fungicide | Oxazole | >63 (D) | Nontoxic | Emulsifiable Concentrate | Liquid Solutions | 36.4 [100g+106.7g  fluzilazole/L] (D) | Nontoxic |
| Famoxadone | Fungicide | Oxazole | >63 (D) | Nontoxic | Water-dispersible Granules | Solid for Direct Use | >200 [22.5% +30% cymoxanil WG](D) | Nontoxic |
| Fenamidone | Fungicide | Imidazole | >159.8 (A, D) | Nontoxic | Water-dispersible Granules | Solid for Direct Use | >118 [Vitera  Flash 4.44%  +Fosetyl Al WG] (D) | Nontoxic |
| Fenazaquin | Miticide | Unknown | 10, 7.3 (A),  7.35 (D) | Moderately Toxic |  |  |  | No Data |
| Fenbuconazole | Fungicide | Azole |  | No Data |  |  |  | No Data |
| Fenhexamid | Fungicide | Anilide | >102 (D) | Nontoxic |  |  |  | No Data |
| Fenoxaprop-P- ethyl | Herbicide | Aryloxyphenoxy propionic acid | >191.4 (A),  >199, >1000 (D) | Nontoxic | Emulsion, oil-in- water | Liquid Emulsions | 23.2 [Puma S 69 EW] (D) | Nontoxic |
| Fenoxycarb | Insecticide | Other carbamate, JH mimic | >204 (D) | Nontoxic |  |  |  | No Data |
| Fenpropidin | Fungicide | Unclassified | >10 (D) | Inconclusive | Emulsifiable Concentrate | Liquid Solutions | 99.9 [Tern 750EC] (D) | Nontoxic |
| Fenpropimorph | Fungicide | Morpholine | >95.6 (A, D) | Nontoxic | Emulsifiable Concentrate | Liquid Solutions | >79.5 [Corbel 750EC] (D) | Nontoxic |
| Fenpyroximate | Miticide | Pyrazole |  | No Data |  |  |  | No Data |
| Fipronil | Insecticide | Pyrazole | 0.00417 (D) | Highly Toxic |  |  |  | No Data |
| Flazasulfuron | Herbicide | Sulfonylurea | >100 (A, D) | Nontoxic | Water-dispersible Granules | Solid for Direct Use | >178.7 [WG 25%] (D) | Nontoxic |
| Flonicamid | Insecticide | Unclassified | 13.6 (A),  >60.5 (A, D) | Nontoxic | Water-dispersible Granules | Solid for Direct Use | >53.3 [50% WG] (D) | Nontoxic |
| Florasulam | Herbicide | Triazolopyrimidine | >100 (D) | Nontoxic | Aqueous Suspension | Liquid Suspensions | >70.25 [50g/L,  Primus] (D) | Nontoxic |
| Fluazifop-P-butyl | Herbicide | Aryloxyphenoxy propionic acid | >200 (D) | Nontoxic | Emulsifiable Concentrate | Liquid Solutions | >382 [EC125g/l] (D) | Nontoxic |
| Fluazinam | Fungicide | 2,6-Dinitroaniline | >100 (A),  >200 (D) | Nontoxic |  |  |  | No Data |

| **TGAI** | **Pesticide Type** | **Chemical Class** | **Mined TGAI LD_50_ (µg a.i./bee)** | **TGAI**  **Toxicity Classification** | **TEP Type** | **Super -TEP** | **Mined TEP LD_50_ (µg a.i./bee)** | **TEP Toxicity Classification** |
| --- | --- | --- | --- | --- | --- | --- | --- | --- |
| Fludioxonyl | Fungicide | Unclassified | >100 (D) | Nontoxic |  |  |  | No Data |
| Flufenacet | Herbicide | Anilide | >170, >329 (D) | Nontoxic |  |  |  | No Data |
| Flumioxazin | Herbicide | N-  phenylphtalamides | >100 (D) | Nontoxic | Wettable Powders | Solid for Direct Use | >200 [Pledge WP50%] (D) | Nontoxic |
| Fluopyram | Fungicide | Amide | >214 (A),  >102.3 (A, D) | Nontoxic | Aqueous Suspension | Liquid Suspensions | [>214µg/bee, Fluopyram SC 500] (D) | Nontoxic |
| Fluoxastrobin | Fungicide | Strobin | >843 (D) | Nontoxic | Emulsifiable Concentrate | Liquid Solutions | 14.4, 25.5  [100EC] (D) | Nontoxic |
| Flupyrsulfuron methyl | Herbicide | Sulfonylurea | >30 (D) | Nontoxic |  |  |  | No Data |
| Fluquinconazole | Fungicide | Azole | >100 (D) | Nontoxic |  |  |  | No Data |
| Flurochloridone | Herbicide | Unknown | >100 (D) | Nontoxic |  |  |  | No Data |
| Fluroxypyr | Herbicide | Pyridinecarboxylic acid |  | No Data | Emulsifiable Concentrate | Liquid Solutions | 208.7 [259g/l EC] (D) | Nontoxic |
| Flurtamone | Herbicide | Unclassified | >304 (D) | Nontoxic |  |  |  | No Data |
| Flusilazole | Fungicide | Azole | 33.75 (D) | Nontoxic |  |  |  | No Data |
| Flutolanil | Fungicide | Anilide | >208.7 (D) | Nontoxic |  |  |  | No Data |
| Flutriafol | Fungicide | Azole | >2 (D) | Inconclusive |  |  |  | No Data |
| Folpel | Fungicide | Thiophthalimide | >236 (D) | Nontoxic | Water-dispersible Granules | Solid for Direct Use | >179 [Folpan 80WDG] (D) | Nontoxic |
| Foramsulfuron | Herbicide | Sulfonylurea | >163.09 (A, D) | Nontoxic | Aqueous Suspension | Liquid Suspensions | >226.3 [Equip SC, 22.5g/l +isoxadife n-ethyl] (D) | Nontoxic |
| Formetanate | Insecticide | N-Methyl  Carbamate | 0.16 (D) | Highly Toxic |  |  |  | No Data |
| Fosetyl-Al | Fungicide | Organophosphate | >140, 462 (D) | Nontoxic | Water-dispersible Granules | Solid for Direct Use | >440 [880g/kg,  Aliette], >118 [Vitera, +fenamidone] (D) | Nontoxic |
| Fosthiazate | Nematicide | Organophosphate | 0.61 (D) | Highly Toxic |  |  |  | No Data |
| Gamma Cyhalothrin | Insecticide | Pyrethroid | 4.2 (D) | Moderately Toxic | Capsule Suspension | Liquid Suspensions | 1.26 [150g/l] (D) | Highly Toxic |
| Glufosinate ammonium | Herbicide | Phosphonoglycine | >600 (D) | Nontoxic |  |  |  | No Data |

| **TGAI** | **Pesticide Type** | **Chemical Class** | **Mined TGAI LD_50_ (µg a.i./bee)** | **TGAI**  **Toxicity Classification** | **TEP Type** | **Super -TEP** | **Mined TEP LD_50_ (µg a.i./bee)** | **TEP Toxicity Classification** |
| --- | --- | --- | --- | --- | --- | --- | --- | --- |
| Glyphosate | Herbicide | Phosphonoglycine | >62.13 (A),  >100 (A, D) | Nontoxic | Soluble Concentrate | Liquid Simple Solutions | >100 [SL 360g/L] (D) | Nontoxic |
| Haloxyfop-R | Herbicide | Aryloxyphenoxy propionic acid | >100 (B, D) | Nontoxic | Emulsifiable Concentrate | Liquid Solutions | 56 [EF-1400] (B, D) | Nontoxic |
| Hexythiazox | Miticide | Unclassified | >112.2 (D) | Nontoxic |  |  |  | No Data |
| Hymexazol | Fungicide | Unknown |  | No Data |  |  |  | No Data |
| Imazalil | Fungicide | Azole | 35.1 (C, D) | Nontoxic |  |  |  | No Data |
| Imazamox | Herbicide | Imidazolinone | >40 (B, C, D) | Nontoxic | Soluble Concentrate | Liquid Simple Solutions | [>500µg/bee product, 40g/L SL] (B) | Nontoxic |
| Imazaquin | Herbicide | Imidazolinone | >6.5 (D) | Inconclusive |  |  |  | No Data |
| Imazosulfuron | Fungicide | Sulfonylurea | >41.6 (B)  112.8 (C) | Nontoxic | Water-dispersible Granules | Solid for Direct Use | >112.8 [50WG] (B) | Nontoxic |
| Imidacloprid | Insecticide | Neonicotinoid | 0.0037 (B, D) | Highly Toxic | Soluble Concentrate | Liquid Simple Solutions | 0.0056 [SL] (B) | Highly Toxic |
| Indoxacarb | Insecticide | Unclassified | 0.194 (D) | Highly Toxic | Water-dispersible Granules | Solid for Direct Use | 23.3 [60%WG] (D) | Nontoxic |
| Iodosulfuron | Herbicide | Sulfonylurea | >80 (D)  >107.6 (B) | Nontoxic | Water-dispersible Granules | Solid for Direct Use | >450.4 [Hussar 20WG] (D) | Nontoxic |
| Ioxynil Octanoate | Herbicide | Hydroxybenzonitrile | >3.27 (D) | Inconclusive |  |  |  | No Data |
| Ioxynil Phenol | Herbicide | Hydroxybenzonitrile | 10.1 (D) | Moderately Toxic |  |  |  | No Data |
| Ipconazole | Fungicide | Azole | >100 (D) | Nontoxic |  |  |  | No Data |
| Iprodione | Fungicide | Dicarboximide | >25 (C, D) | Nontoxic |  |  |  | No Data |
| Iprovalicarb | Fungicide | Carbamate | >199 (B, C, D) | Nontoxic |  |  |  | No Data |
| Iron sulphate | Herbicide | Inorganic |  | No Data |  |  |  | No Data |
| Isoproturon | Herbicide | Urea | 195 (D) | Nontoxic | Aqueous Suspension | Liquid Suspensions | >99.2 [500SC] (B) | Nontoxic |
| Isopyrazam | Fungicide | Pyrazole | >192.27,  >95.5 (D) | Nontoxic | Emulsifiable Concentrate | Liquid Solutions | 230.9 [125 g/L] (B) | Nontoxic |
| Isoxaben | Herbicide | Benzamide | >100 (B, D) | Nontoxic | Aqueous Suspension | Liquid Suspensions | >100 [500SC] (B) | Nontoxic |
| Isoxaflutole | Herbicide | Oxazole | >108.9 (B)  >168.7 (C, D) | Nontoxic |  |  |  | No Data |
| Kresoxim-methyl | Fungicide | Strobin | >110 (D) | Nontoxic |  |  |  | No Data |

| **TGAI** | **Pesticide Type** | **Chemical Class** | **Mined TGAI LD_50_ (µg a.i./bee)** | **TGAI**  **Toxicity Classification** | **TEP Type** | **Super -TEP** | **Mined TEP LD_50_ (µg a.i./bee)** | **TEP Toxicity Classification** |
| --- | --- | --- | --- | --- | --- | --- | --- | --- |
| lambda- Cyhalothrin | Insecticide | Pyrethroid | 0.91 (B, D) | Highly Toxic | Emulsifiable Concentrate | Liquid Solutions | 0.118 [50EC] (B) | Highly Toxic |
| lambda- Cyhalothrin | Insecticide | Pyrethroid | 0.91 (B, D) | Highly Toxic | Capsule Suspension | Liquid Suspensions | 8.5 [100CS] (B) | Moderately Toxic |
| Lenacil | Herbicide | Uracil |  | No Data | Wettable Powders | Solid for Direct Use | >100 [800 g/kg WP] (B) | Nontoxic |
| Linuron | Herbicide | Urea | >160 (D) | Nontoxic |  |  |  | No Data |
| Lufenuron | Insecticide | Benzoylurea | >197 (B) >38  (D) | Nontoxic |  |  |  | No Data |
| Malathion | Insecticide | Organophosphate |  | No Data | Emulsion, oil-in- water | Liquid Emulsions | 0.4 [440EW] (D) | Highly Toxic |
| Mancozeb | Fungicide | Dithiocarbamate | 140.6 (B, D) | Nontoxic |  |  |  | No Data |
| Mandipropamid | Fungicide | Amide | >200 (D) | Nontoxic | Aqueous Suspension | Liquid Suspensions | >215 [250 g/L SC] (D) | Nontoxic |
| Maneb | Fungicide | Dithiocarbamate | >89.49 (D) | Nontoxic |  |  |  | No Data |
| MCPA | Herbicide | Chlorophenoxy acid | 200 (D) | Nontoxic |  |  |  | No Data |
| MCPB | Herbicide | Chlorophenoxy acid | >81.83 (D) | Nontoxic |  |  |  | No Data |
| Mecoprop | Herbicide | Chlorophenoxy acid | >100 (D) | Nontoxic |  |  |  | No Data |
| Mecoprop-P | Herbicide | Chlorophenoxy acid | >100 (B, D) | Nontoxic |  |  |  | No Data |
| Mepanipyrim | Fungicide | Anilinopyrimidine | >100 (D) | Nontoxic | Wettable Powders | Solid for Direct Use | >51.1 [>100 µg/bee product, 50%WP] (D) | Nontoxic |
| Meptyldinocap | Fungicide | Dinitrophenol |  | No Data | Emulsifiable Concentrate | Liquid Solutions | 90 [34.7% EC, GF1478] (D) | Nontoxic |
| Mesosulfuron | Herbicide | Sulfonylurea | >105.6 (B) 5.6 (D) | Inconclusive |  |  |  | No Data |
| Mesotrione | Herbicide | Benzoylcyclohexane dione | >11 (B, D) | Inconclusive | Aqueous Suspension | Liquid Suspensions | 80.5 [100 g/L SC] (B) | Nontoxic |
| Metalaxyl | Fungicide | Phenylamide | >97.3 (B) >25 (D) | Nontoxic | Emulsifiable Concentrate | Liquid Solutions | >127 [480 EC] (D) | Nontoxic |
| Metaldehyde | Mulluscicide | Aldehyde | >87.5 (B, D) | Nontoxic |  |  |  | No Data |
| Metamitron | Herbicide | Triazinone | >97.2 (D) | Nontoxic | Aqueous  Suspension | Liquid  Suspensions | 123.3 [700 SC] (D) | Nontoxic |
| Metazachlor | Herbicide | Chloroacetanilide | >72.9 (D) | Nontoxic | Aqueous Suspoemulsion | Liquid Dual Character | >92.12 [500 SE] | Nontoxic |
| Metconazole | Fungicide | Azole | 85 (B, D) | Nontoxic |  |  |  | No Data |

| **TGAI** | **Pesticide Type** | **Chemical Class** | **Mined TGAI LD_50_ (µg a.i./bee)** | **TGAI**  **Toxicity Classification** | **TEP Type** | **Super -TEP** | **Mined TEP LD_50_ (µg a.i./bee)** | **TEP Toxicity Classification** |
| --- | --- | --- | --- | --- | --- | --- | --- | --- |
| Methiocarb | Insecticide | Methyl Carbamate | 0.47 (D) | Highly Toxic |  |  |  | No Data |
| Methomyl | Insecticide | Carbamate | 0.28 (B) 0.2 (D) | Highly Toxic | Soluble Concentrate | Liquid Simple Solutions | 0.2 [20 SL] (B) | Highly Toxic |
| Methoxyfenozide | Insecticide | Diacylhydrazine | >100 (D) | Nontoxic | Aqueous Suspension | Liquid Suspensions | [>289µg/bee product, 240 SC] (D) | Nontoxic |
| Metiram zinc | Fungicide | Dithiocarbamate | >80 (D) | Nontoxic |  |  |  | No Data |
| Metosulam | Herbicide | Triazolopyrimidine | >106 (B) >50 (D) | Nontoxic | Aqueous Suspension | Liquid Suspensions | [>270µg/bee product, 100 g/L] (B) | Nontoxic |
| Metrafenone | Fungicide | Benzophenone | >114 (D) | Nontoxic | Aqueous Suspension | Liquid Suspensions | >32.7 [300 SC],  >58.3 [500 SC] (D) | Nontoxic |
| Metribuzin | Herbicide | Triazinone | 166 (D) | Nontoxic | Water-dispersible Granules | Solid for Direct Use | 37.1 [Metribuzin 70WG], 134 [Mistral 700 WG] (D) | Nontoxic |
| Metsulfuron- methyl | Herbicide | Sulfonylurea | >25 (B), >44.3 (D) | Nontoxic | Water-soluble Granules | Solid for Direct Use | >113.7 [20SG] (B) | Nontoxic |
| Milbemectin | Insecticide | Macrocyclic Lactone | 0.4 (D) | Highly Toxic | Emulsifiable Concentrate | Liquid Solutions | >0.0423  [4.23µg product/bee, EC 1%] (D) | Inconclusive |
| Myclobutanil | Fungicide | Azole |  | No Data | Emulsion, oil-in- water | Liquid Emulsions | 33.9 [200 g/L] (B) | Nontoxic |
| Napropamide | Herbicide | Amide | 121 (D) | Nontoxic | Aqueous Suspension | Liquid Suspensions | >100 [Devrinol 450 SC] (B, D) | Nontoxic |
| Nicosulfuron | Herbicide | Sulfonylurea | >1000 ppm (D) | Nontoxic | Aqueous Suspension | Liquid Suspensions | >5.24 [>131 µg/bee product, SL-950 4% SC] (D) | Inconclusive |
| Oryzalin | Herbicide | 2,6-Dinitroaniline |  | No Data | Emulsifiable Concentrate | Liquid Solutions | 32 [480 g/L] (D) | Nontoxic |
| Oxadiargyl | Herbicide | Unclassified | >200 (D) | Nontoxic |  |  |  | No Data |
| Oxadiazon | Herbicide | Oxidiazole | >110.5 (B)  >400 (D) | Nontoxic | Emulsifiable Concentrate | Liquid Solutions | >51.5 [250 g/L] (B) | Nontoxic |
| Oxamyl | Insecticide | Carbamate | 0.38 (D) | Highly Toxic | Soluble Concentrate | Liquid Simple Solutions | 0.26 [10% SL] (D) | Highly Toxic |

| **TGAI** | **Pesticide Type** | **Chemical Class** | **Mined TGAI LD_50_ (µg a.i./bee)** | **TGAI**  **Toxicity Classification** | **TEP Type** | **Super -TEP** | **Mined TEP LD_50_ (µg a.i./bee)** | **TEP Toxicity Classification** |
| --- | --- | --- | --- | --- | --- | --- | --- | --- |
| Oxasulfuron | Herbicide | Sulfonylurea | >100 (D) | Nontoxic | Water-dispersible Granules | Solid for Direct Use | >741 [75WG] (D) | Nontoxic |
| Oxathiapiprolin | Fungicide | Piperidinyl thiazole isoxazoline | >40.26 (B) | Nontoxic | Oil-Based Suspension | Liquid Suspensions | >137.44 [100 g/L] (B) | Nontoxic |
| Oxyfluorfen | Herbicide | Diphenyl Ether | >100 (B, D) | Nontoxic |  |  |  | No Data |
| Penconazole | Fungicide | Azole | >112 (D),  >186 | Nontoxic | Emulsifiable Concentrate | Liquid Solutions | [>112µg product/bee, Topas 10EC] (B) | Nontoxic |
| Pencycuron | Fungicide | Urea | >98.5 (B, D) | Nontoxic |  |  |  | No Data |
| Pendimethalin | Herbicide | 2,6-Dinitroaniline | >101.2 (B, D) | Nontoxic | Aqueous Suspension | Liquid Suspensions | >198.5 [400SC] (B) | Nontoxic |
| Pendimethalin | Herbicide | 2,6-Dinitroaniline | >101.2 (B, D) | Nontoxic | Capsule Suspension | Liquid Suspensions | [311µg/bee  product, 455 g/L] (B) | Nontoxic |
| Penoxsulam | Herbicide | Triazolopyrimidine | >100 (D) | Nontoxic | Oil-Based Suspension | Liquid Suspensions | [>160µg product/bee, 20.4 g/L] (B) | Nontoxic |
| Penthiopyrad | Fungicide | Amide | >500 (B) | Nontoxic | Emulsifiable Concentrate | Liquid Solutions | 50.7 [200 g/L] (B) | Nontoxic |
| Penthiopyrad | Fungicide | Amide | >500 (B) | Nontoxic | Aqueous Suspension | Liquid Suspensions | >107.2 [200 g/L] (B) | Nontoxic |
| Pethoxamid | Herbicide | Amide | 200 (D) | Nontoxic |  |  |  | No Data |
| Phenmedipham | Herbicide | Bis-Carbamate | 23 (D) | Nontoxic |  |  |  | No Data |
| Phosmet | Insecticide | Organophosphate | 0.37 (B, D) | Highly Toxic |  |  |  | No Data |
| Picloram | Herbicide | Pyridinecarboxylic acid | >100 (D) | Nontoxic |  |  |  | No Data |
| Picolinafen | Herbicide | Pyridinecarboxylic acid | >200 (B, D) | Nontoxic | Water-dispersible Granules | Solid for Direct Use | >150 [Picosolo, 750 g/kg] (B, D) | Nontoxic |
| Picoxystrobin | Fungicide | Strobin |  | No Data | Aqueous Suspension | Liquid Suspensions | >200 [250 SC] (B, D) | Nontoxic |
| Pinoxaden | Herbicide | Unclassified | >200 (B, D) | Nontoxic | Emulsifiable Concentrate | Liquid Solutions | 9.05 [A12303 C] (B) | Moderately Toxic |
| Pirimicarb | Insecticide | Carbamate | 4 (B, D) | Moderately Toxic | Water-dispersible Granules | Solid for Direct Use | 14 [500 WG] (B, D) | Nontoxic |
| Pirimiphos-methyl | Insecticide | Organophosphate |  | No Data |  |  |  | No Data |

| **TGAI** | **Pesticide Type** | **Chemical Class** | **Mined TGAI LD_50_ (µg a.i./bee)** | **TGAI**  **Toxicity Classification** | **TEP Type** | **Super -TEP** | **Mined TEP LD_50_ (µg a.i./bee)** | **TEP Toxicity Classification** |
| --- | --- | --- | --- | --- | --- | --- | --- | --- |
| Potassium phosphonate | Fungicide | Inorganic | >145 (B)  >50.34 (D) | Nontoxic |  |  |  | No Data |
| Prochloraz | Fungicide | Azole | >101.06 (B, D) | Nontoxic | Emulsion, oil-in- water | Liquid Emulsions | 27.4[450 g/L] (B) | Nontoxic |
| Propamocarb | Fungicide | Carbamate | >84 (B, D) | Nontoxic |  |  |  | No Data |
| Propaquizafop | Herbicide | Aryloxyphenoxy propionic acid | >20 (D) | Nontoxic | Emulsifiable Concentrate | Liquid Solutions | >18.9 [>189 µg/bee product, 100 g/L EC] (D) | Nontoxic |
| Propiconazole | Fungicide | Azole | >100 (D) | Nontoxic |  |  |  | No Data |
| Propineb | Fungicide | Dithiocarbamate |  | No Data | Water-dispersible Granules | Solid for Direct Use | >70 [700 g/kg] (D) | Nontoxic |
| Propoxycarbazone | Herbicide | Triazolone | >319 (D) | Nontoxic | Water-dispersible Granules | Solid for Direct Use | >319 [70% WG] (D) | Nontoxic |
| Propyzamide | Herbicide | Amide |  | No Data |  |  |  | No Data |
| Proquinazid | Fungicide | Quinazolinone | >125 (D) | Nontoxic | Emulsifiable Concentrate | Liquid Solutions | >99.75 [200 g/L] (D) | Nontoxic |
| Prosulfocarb | Herbicide | Thiocarbamate |  | No Data | Emulsifiable Concentrate | Liquid Solutions | 103.4 [80EC, SF245] (B, D) | Nontoxic |
| Prosulfuron | Herbicide | Sulfonylurea | >100 (B) | Nontoxic | Water-dispersible Granules | Solid for Direct Use | >112.5 [750 g/kg]  (B), 62 [Eclat 63WG] (D) | Nontoxic |
| Prothioconazole | Fungicide | Azole | >71 (D) | Nontoxic | Emulsifiable Concentrate | Liquid Solutions | >48.7 [250 g/L] (D) | Nontoxic |
| Pymetrozine | Insecticide | Pyridine | >117 (B, D) | Nontoxic | Water-dispersible Granules | Solid for Direct Use | [171µg/bee product, 500 g/kg] (B) | Nontoxic |
| Pyraclostrobin | Fungicide | Strobin | >73.1 (D) | Nontoxic | Emulsifiable Concentrate | Liquid Solutions | >79.9 [250g/L] (D) | Nontoxic |
| Pyraflufen-ethyl | Herbicide | Phenylpyrazole | >112 (B, D) | Nontoxic | Emulsifiable Concentrate | Liquid Solutions | 12.42 [2.5% EC] (B) | Nontoxic |
| Pyrethrin | Insecticide | Pyrethroid | 0.022 (D) | Highly Toxic | Emulsifiable Concentrate | Liquid Solutions | 0.95 [18.61 g/L,  Pyrevert] (B) | Highly Toxic |
| Pyridaben | Insecticide | Unclassified | 0.535 (B) | Highly Toxic | Wettable Powders | Solid for Direct Use | 2.12 [750 g/kg] (B) | Moderately Toxic |
| Pyridaben | Insecticide | Unclassified | 0.535 (B) | Highly Toxic | Aqueous Suspension | Liquid Suspensions | 4.45 [SC] (B) | Moderately Toxic |

| **TGAI** | **Pesticide Type** | **Chemical Class** | **Mined TGAI LD_50_ (µg a.i./bee)** | **TGAI**  **Toxicity Classification** | **TEP Type** | **Super -TEP** | **Mined TEP LD_50_ (µg a.i./bee)** | **TEP Toxicity Classification** |
| --- | --- | --- | --- | --- | --- | --- | --- | --- |
| Pyridate | Herbicide | Phenylpyridazine | >109.8 (B)  >100 (D) | Nontoxic | Wettable  Powders | Solid for Direct  Use | >105.8 [450 g/kg]  (B) | Nontoxic |
| Pyrimethanil | Fungicide | Pyrimidine | >100 (D) | Nontoxic | Aqueous  Suspension | Liquid  Suspensions | >200 [400SC] (D) | Nontoxic |
| Pyriofenone | Fungicide | Aryl phenyl ketone | >100 (B, D) | Nontoxic |  |  |  | No Data |
| Pyriproxyfen | Insecticide | Unclassified |  | No Data | Emulsifiable Concentrate | Liquid Solutions | 74 [10EC] (B) | Nontoxic |
| Pyroxsulam | Herbicide | Triazolopyrimidine | >107.4 (B, D) | Nontoxic | Water-dispersible Granules | Solid for Direct Use | >104 [75WG] (B) | Nontoxic |
| Quinmerac | Herbicide | Quinolinecarboxylic acid | >100 (D)  >108.51 (B) | Nontoxic |  |  |  | No Data |
| Quinoclamine | Herbicide | Unclassified |  | No Data | Wettable Powders | Solid for Direct Use | >28, > 43  [Mogeton 25WP]  (B, D) | Nontoxic |
| Quinoxyfen | Fungicide | Quinoline | >100 (B, D) | Nontoxic | Aqueous Suspension | Liquid Suspensions | >100 [500SC] (B) | Nontoxic |
| Quizalofop-P | Herbicide | Aryloxyphenoxy propionic acid |  | No Data |  |  |  | No Data |
| Quizalofop-P ethyl | Herbicide | Aryloxyphenoxy propionic acid | >100 (B, D) | Nontoxic | Emulsifiable Concentrate | Liquid Solutions | 10.4 [50g/L,  Targa Super] (B, D) | Moderately Toxic |
| Quizalofop-P tefuryl | Herbicide | Aryloxyphenoxy propionic acid |  | No Data | Emulsifiable Concentrate | Liquid Solutions | 16.8 [40g/L] (B, D) | Nontoxic |
| Rimsulfuron | Herbicide | Sulfonylurea | >100 (B, D) | Nontoxic | Water-dispersible Granules | Solid for Direct Use | 41.1 [25  WG+surfactant Excell] (B, D) | Nontoxic |
| Sedaxane | Fungicide | Pyrazole | > 4 (B, D) | Inconclusive | Suspension for Seeds | Liquid Suspensions | >46.9 [>109.6 µg/bee product, 500g/L, A16148F] (B, D) | Nontoxic |
| Silthiofam | Fungicide | Unclassified |  | No Data |  |  |  | No Data |
| Sintofen |  | Unclassified |  | No Data | Soluble Concentrate | Liquid Simple Solutions | > 100 [Croisor 100 g/L] (B, D) | Nontoxic |
| S-metolachlor | Herbicide | Chloroacetanilide | >100 (A) > 85 (C, D) | Nontoxic | Emulsifiable Concentrate | Liquid Solutions | 160 [960 g/l EC] (D) | Nontoxic |
| Spinosad | Insecticide | Macrocyclic Lactone | 0.057 (C, D) | Highly Toxic | Aqueous Suspension | Liquid Suspensions | 0.049 [480 g/L] (C) | Highly Toxic |

| **TGAI** | **Pesticide Type** | **Chemical Class** | **Mined TGAI LD_50_ (µg a.i./bee)** | **TGAI**  **Toxicity Classification** | **TEP Type** | **Super -TEP** | **Mined TEP LD_50_ (µg a.i./bee)** | **TEP Toxicity Classification** |
| --- | --- | --- | --- | --- | --- | --- | --- | --- |
| Spirodiclofen | Insecticide | Tetramic acid | >196 (B, D) | Nontoxic | Aqueous  Suspension | Liquid  Suspensions | >100 [240 g/L] (B,  D) | Nontoxic |
| Spirotetramat | Insecticide | Tetramic acid | >107.3 (B, D) | Nontoxic | Oil-Based Suspension | Liquid Suspensions | 91.7 [150 g/L] (B) | Nontoxic |
| Spiroxamine | Fungicide | Morpholine | > 100 (A, B, D) | Nontoxic | Emulsifiable Concentrate | Liquid Solutions | [>12.5, 77 µg/bee  product, 500 g/L] (B) | Nontoxic |
| Sulcotrione | Herbicide | Benzoylcyclohexane dione | >50 (B, D) | Nontoxic | Aqueous Suspension | Liquid Suspensions | [>763µg/bee  product, 300 g/L] (B) | Nontoxic |
| Sulfosulfuron | Herbicide | Sulfonylurea | >30 (A, B, D) | Nontoxic | Water-dispersible Granules | Solid for Direct Use | >104 [800 g/kg] (B) | Nontoxic |
| Sulfur | Fungicide | Inorganic |  | No Data | Water-dispersible Granules | Solid for Direct Use | > 100 [80% WG] (B) | Nontoxic |
| Sulfur | Fungicide | Inorganic |  | No Data | Others | Liquid Suspensions | > 106.8 [dust] (B) | Nontoxic |
| Tau-fluvalinate | Miticide | Pyrethroid | 12.6 (B, D) | Nontoxic | Emulsion, oil-in- water | Liquid Emulsions | 100 [240 g/l EW] (B, D) | Nontoxic |
| Tebuconazole | Fungicide | Azole | >83.05 (B, D) | Nontoxic | Emulsion, oil-in- water | Liquid Emulsions | > 187 [Folicur  EW, 250 g/L] (B, D) | Nontoxic |
| Tebufenozide | Insecticide | Diacylhydrazine (molting hormone agonist) |  | No Data | Aqueous Suspension | Liquid Suspensions | >100 [240 g/L] (B) | Nontoxic |
| Tebufenpyrad | Miticide | Pyrazole | 60.3 (B, D) | Nontoxic | Wettable Powders | Solid for Direct Use | 6.4, 8 [32, 40  µg/bee product, 200 g/kg] (B, D) | Moderately Toxic |
| Teflubenzuron | Insecticide | Benzoylurea | 72 (B) | Nontoxic | Aqueous  Suspension | Liquid  Suspensions | 110 [150 g/L] (B) | Nontoxic |
| Tefluthrin | Insecticide | Pyrethroid | 0.68 (B,D) | Highly Toxic |  |  |  | No Data |
| Tembotrione | Herbicide | Benzoylcyclohexane dione | > 97.8 (A);  >92.8 (B, D) | Nontoxic | Oil-Based Suspension | Liquid Suspensions | 14 [44 g/L] (B),  328 [oily  Suspension 44 g/L] (D) | Nontoxic |
| Tepraloxydim | Herbicide | Cyclohexenone derivative | > 200 (C, D) | Nontoxic | Emulsifiable Concentrate | Liquid Solutions | >200 [200 g/L  BAS 620 00 H+  Dash HC EC] (D) | Nontoxic |
| Tetraconazole | Fungicide | Azole | >130 (B, D) | Nontoxic | Microemulsion | Liquid  Emulsions | 16.3 [40 g/L] (B) | Nontoxic |

| **TGAI** | **Pesticide Type** | **Chemical Class** | **Mined TGAI LD_50_ (µg a.i./bee)** | **TGAI**  **Toxicity Classification** | **TEP Type** | **Super -TEP** | **Mined TEP LD_50_ (µg a.i./bee)** | **TEP Toxicity Classification** |
| --- | --- | --- | --- | --- | --- | --- | --- | --- |
| Thiabendazole | Fungicide | Benzimidazole | >4 | Inconclusive | Aqueous Suspension | Liquid Suspensions | >200 [500 g/l SC]  (B), >212 [Tecto 500SC] (D) | Nontoxic |
| Thiacloprid | Insecticide | Neonicotinoid | 17.32 (A, C, D); 12.8 (A) | Nontoxic | Aqueous Suspension | Liquid Suspensions | [8.51µg/bee product, 480 g/L] (C, D) | Moderately Toxic |
| Thiamethoxam | Insecticide | Neonicotinoid | 0.005 (B, D) | Highly Toxic | Others | Liquid Suspensions | 0.00936 [Dust  from formulation A9700B (dust contained 7.24% thiamethoxam)] (B) | Highly Toxic |
| Thiencarbazone- methyl | Herbicide | Triazolone | >199 (A, B),  >200 (D) | Nontoxic | Aqueous Suspension | Liquid Suspensions | >60.6 [>316µg/bee product, 450 g/L] (B) | Nontoxic |
| Thifensulfuron- methyl | Herbicide | Sulfonylurea | >100 (C, D) | Nontoxic |  |  |  | No Data |
| Thiophanate- methyl | Fungicide | Benzimidazole precursor | >100 (C, D) | Nontoxic |  |  |  | No Data |
| Thiram | Fungicide | Dithiocarbamate | >100 (C, D) | Nontoxic |  |  |  | No Data |
| Tolclofos-methyl | Fungicide | Unclassified |  | No Data |  |  |  | No Data |
| Tralkoxydim | Herbicide | Cyclohexenone derivative | 54 (D) | Nontoxic | Aqueous Suspension | Liquid Suspensions | >117 [242 g/L] (B) | Nontoxic |
| Triadimenol | Fungicide | Azole | >224.8 (B) | Nontoxic | Emulsifiable Concentrate | Liquid Solutions | 64.4 [250 g/L] (B) | Nontoxic |
| Triadimenol | Fungicide | Azole | >224.8 (B) | Nontoxic | Suspension for Seeds | Liquid Suspensions | 298 [94 g/L] (B) | Nontoxic |
| Triallate | Herbicide | Thiocarbamate |  | No Data |  |  |  | No Data |
| Triasulfuron | Herbicide | Sulfonylurea | >100 (B, D) | Nontoxic | Water-dispersible Granules | Solid for Direct Use | >207 [200 g/kg] (B) | Nontoxic |
| Tribenuron | Herbicide | Sulfonylurea | >9.1 (B, D) | Inconclusive | Water-dispersible Granules | Solid for Direct Use | >186 [750 g/kg] (B, D) | Nontoxic |
| Triclopyr | Herbicide | Pyridine | >100 (B, D) | Nontoxic | Emulsifiable Concentrate | Liquid Solutions | >100 [480 g/L] (B,D) | Nontoxic |

| **TGAI** | **Pesticide Type** | **Chemical Class** | **Mined TGAI LD_50_ (µg a.i./bee)** | **TGAI**  **Toxicity Classification** | **TEP Type** | **Super -TEP** | **Mined TEP LD_50_ (µg a.i./bee)** | **TEP Toxicity Classification** |
| --- | --- | --- | --- | --- | --- | --- | --- | --- |
| Trifloxystrobin | Fungicide | Strobin | >200 (C, D) | Nontoxic | Emulsifiable Concentrate | Liquid Solutions | >142 [125 g/L] (C, D) | Nontoxic |
| Trifloxystrobin | Fungicide | Strobin | >200 (C, D) | Nontoxic | Water-dispersible Granules | Solid for Direct Use | >187 [500 g/kg] (C, D) | Nontoxic |
| Triflusulfuron | Herbicide | Sulfonylurea | > 100 (B, D) | Nontoxic |  |  |  | No Data |
| Trinexapac | Plant Growth Regulator | Unclassified | >200 (D) | Nontoxic | Emulsifiable Concentrate | Liquid Solutions | >108 [250 g/L] (B, D) | Nontoxic |
| Triticonazole | Fungicide | Azole | >155.5 (B, D) | Nontoxic |  |  |  | No Data |
| Tritosulfuron | Herbicide | Sulfonylurea | 200 (C, D) | Nontoxic | Water-dispersible Granules | Solid for Direct Use | [121.62µg/bee product, 714 g/kg +adjuvant] (C, D) | Nontoxic |
| Valifenalate | Fungicide | Amino acid | > 106.6 (B,D) | Nontoxic | Water-dispersible Granules | Solid for Direct Use | >200 [6% valifenalate+4 8%folpet WG] (B) | Nontoxic |
| Zeta-cypermethrin | Insecticide | Pyrethroid | 0.11; 0.172;  0.56 (A) [Geomean  0.219] | Highly Toxic | Emulsion, oil-in- water | Liquid Emulsions | 0.044  [0.436µg/bee product, 100g/L]  (B, D) | Highly Toxic |
| Ziram | Fungicide | Dithiocarbamate | >100 (C,D) | Nontoxic |  |  |  | No Data |
| Zoxamide | Fungicide | Benzamide |  | No Data | Wettable Powders | Solid for Direct Use | >200 [80WP, 7-  8% zoxamide +  67-70% mancozeb] (D) | Nontoxic |
| Zoxamide | Fungicide | Benzamide |  | No Data | Water-dispersible Granules | Solid for Direct Use | >153 [75WG,  8.3% Zoxamide +66.7% mancozeb] (C, D) | Nontoxic |
| Zoxamide | Fungicide | Benzamide |  | No Data | Aqueous  Suspension | Liquid  Suspensions | >147 [240 g/L]  (D) | Nontoxic |
